# Advances in the measurement of coverage for RMNCH and nutrition: from contact to effective coverage

**DOI:** 10.1136/bmjgh-2018-001297

**Published:** 2019-06-24

**Authors:** Agbessi Amouzou, Hannah Hogan Leslie, Malathi Ram, Monica Fox, Safia S Jiwani, Jennifer Requejo, Tanya Marchant, Melinda Kay Munos, Lara M E Vaz, William Weiss, Chika Hayashi, Ties Boerma

**Affiliations:** 1 Department of International Health, Bloomberg School of Public Health, Johns Hopkins University, Baltimore, Maryland, USA; 2 Global Health and Population, Harvard TH Chan School of Public Health, Boston, Massachusetts, USA; 3 Data and Analytics Section, Division of Data, Research and Policy, UNICEF USA, New York, New York, USA; 4 Disease Control, London School of Hygiene & Tropical Medicine, London, United Kingdom; 5 Global Health, Save the Children, Fairfield, Connecticut, USA; 6 Community Health Sciences, Max Rady College of Medicine, University of Manitoba, Winnipeg, Manitoba, Canada

**Keywords:** intervention coverage, effective coverage, quality of care, quality-adjusted coverage, continuum of care, RMNCH+N

## Abstract

Current methods for measuring intervention coverage for reproductive, maternal, newborn, and child health and nutrition (RMNCH+N) do not adequately capture the quality of services delivered. Without information on the quality of care, it is difficult to assess whether services provided will result in expected health improvements. We propose a six-step coverage framework, starting from a target population to (1) service contact, (2) likelihood of services, (3) crude coverage, (4) quality-adjusted coverage, (5) user-adherence-adjusted coverage and (6) outcome-adjusted coverage. We support our framework with a comprehensive review of published literature on effective coverage for RMNCH+N interventions since 2000. We screened 8103 articles and selected 36 from which we summarised current methods for measuring effective coverage and computed the gaps between ‘crude’ coverage measures and quality-adjusted measures. Our review showed considerable variability in data sources, indicator definitions and analytical approaches for effective coverage measurement. Large gaps between crude coverage and quality-adjusted coverage levels were evident, ranging from an average of 10 to 38 percentage points across the RMNCH+N interventions assessed. We define effective coverage as the proportion of individuals experiencing health gains from a service among those who need the service, and distinguish this from other indicators along a coverage cascade that make quality adjustments. We propose a systematic approach for analysis along six steps in the cascade. Research to date shows substantial drops in effective delivery of care across these steps, but variation in methods limits comparability of the results. Advancement in coverage measurement will require standardisation of effective coverage terminology and improvements in data collection and methodological approaches.

**Summary box**

- Most reproductive, maternal, newborn, and child health and nutrition (RMNCH+N) intervention coverage indicators—the proportion of the population in need of an intervention that receives it—monitored for decades do not capture the quality of delivery of the interventions and therefore provide only weak links with actual health benefits received by the population in need.
- An increasing number of studies attempt to measure effective coverage indicators that also capture the quality of care and quantify the gaps between crude coverage and quality-adjusted measures.
- Our comprehensive review of the literature shows evidence of large coverage quality gaps in RMNCH+N, but the definitions, terminologies, analytical methodologies used vary widely, limiting the interpretability and comparability of the results.
- Building on previous frameworks and our review of current practices, we propose an organising framework to harmonise terminologies and methodological approaches for the measurement of a coverage cascade, and a definition of effective coverage as ‘the proportion of individuals experiencing health gains from a service among those who need the service’.

## Introduction

Monitoring intervention coverage, defined as the proportion of the population in need of a health intervention who receives it, is essential for tracking progress towards universal health coverage—an aim of Sustainable Development Goal 3. Although the coverage of many interventions along the continuum of care for women’s, children’s and adolescents’ health has increased in the past decade, there is increasing evidence that national coverage indicators may overstate the health benefits of the programme because of poor quality of services.^1 2^

Advancement in coverage measurement requires a shift from tracking ‘crude’ or ‘contact’ coverage to effective coverage, accounting for the quality of services and their impact on people’s health. Crude coverage indicators provide no indication about the quality of interventions, whereas contact coverage simply captures contact with a provider as a proxy for adequate receipt of the needed service. In recent years, an increasing number of studies have quantified the alarming gaps between crude or contact coverage indicators and those that measure the receipt and benefits from high-quality services (effective coverage indicators).^1–5^

The definition and measurement of effective coverage varies between studies. There is a need for standard terminology and methods for coverage measurement. We propose a framework for the measurement of effective coverage, apply it in our systematic review of the literature and provide examples of how the framework can be operationalised for reproductive, maternal, newborn, and child health and nutrition (RMNCH+N).

## A framework for measurement of effective coverage: the coverage cascade

In general, the term effective coverage incorporates not just receipt of services but also their quality. Quality of care comprises several domains traditionally organised into inputs (eg, service availability and whether a provider had access to needed equipment, diagnostics and medicines, referred to as readiness measures), the process of service delivery (eg, whether health providers followed protocols or standards of care) and outcomes, including health benefits as well as patient satisfaction.^6 7^ Effective coverage literature has also included consideration of patient’s adherence to recommended practices or treatment as an indication of quality care. Focusing on better health as the desired outcome of health system functioning, effective coverage has also alternatively been defined as ‘the fraction of potential health gain that is actually delivered to the population through the health system, given its capacity’.^8 9^ Finally, definitions of effective coverage vary across disciplines. For example, for food fortification programmes, effective coverage has been defined in some studies as the proportion of the population in need that uses the product with recommended frequency and quantity.^10^

**Figure 1:**
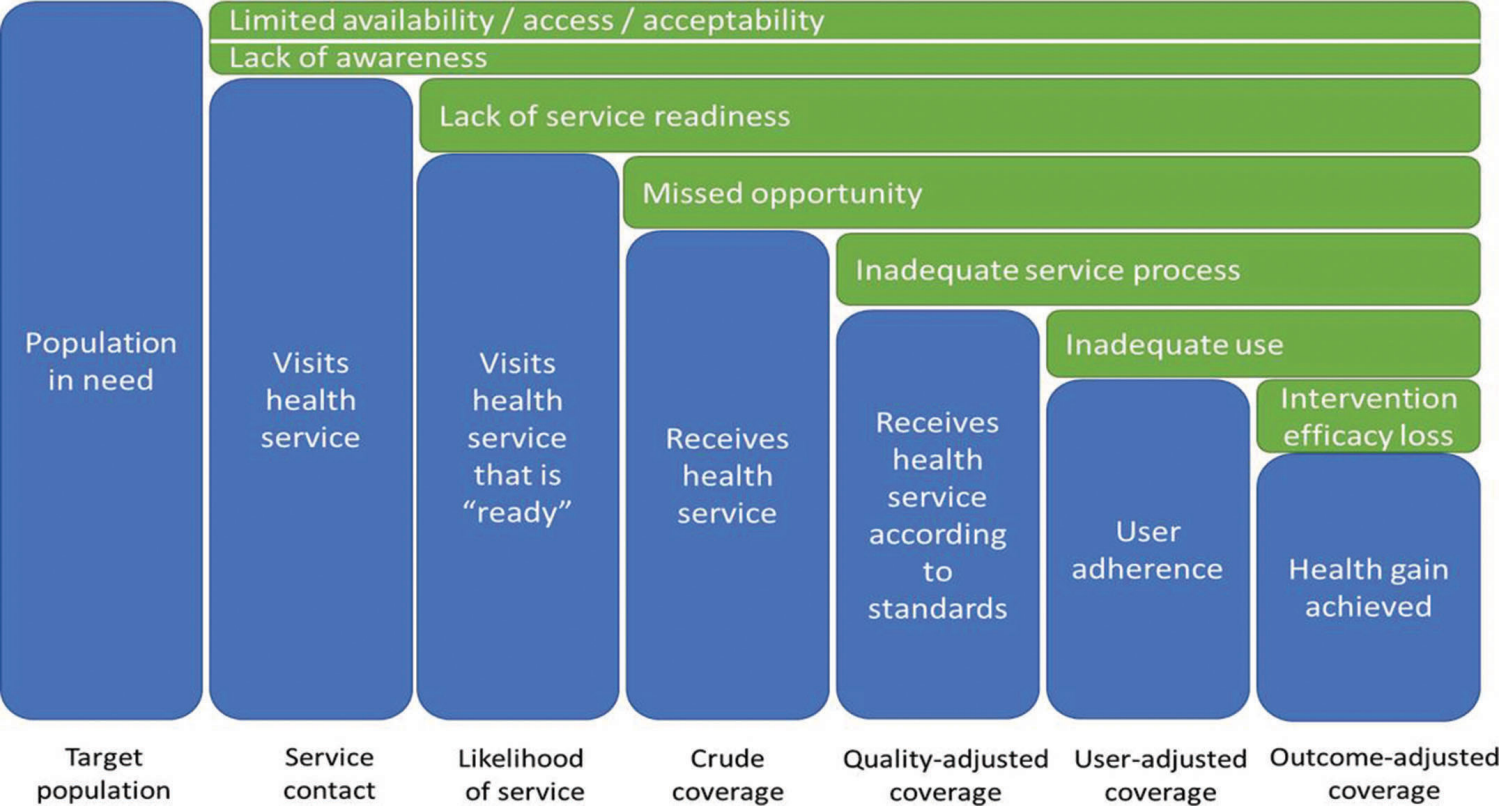
Hypothetical cascade of the potential losses of healthbenefits of interventions among a population in need of a specific healthservice.

Building on the Tanahashi framework, we propose a cascade framework that defines (table 1) and organises the components of coverage in a stepwise fashion (figure 1).^11^ The framework can be used as a standard approach to identify (and quantify) the losses to potential health benefits that can occur at each step and to assess the current measurement practices and gaps for each step. Table 2 presents an illustration of data collection approaches used to capture information on each of the steps of the framework, the types of interventions that can be introduced to address challenges and examples of how to assess each step. While the cascade applies well to a population cohort moving through each step, with the measure of each conditional on the previous, there are exceptions where a step may be successfully realised even though the previous step was not achieved. For example, user adherence can occur even though the service was not provided according to standard. Adherence to a long-term contraceptive method may occur even though counselling during service provision did not follow all standards. Such cases would often be evident at the individual level, but be less evident in population-based aggregate measures of coverage. Consequently, cross-sectional measures of individual steps in the coverage cascade may yield results that are higher than the previous step if the steps are not nested within each other.

**Table 1:** Definition and description of potential loss of each step of the cascade framework

Component	Definition	Potential loss of health benefits
Target population	All who need a service/intervention	
Service contact	Proportion among those in need (the target population) who visit a health service.	Service access, awareness of services and service acceptability. Lack of access to services can occur because of structural (eg, facilities are too far or not open), financial or other obstacles. Individuals may be unaware of the need for care or that services exist for the conditions they have (eg, asymptomatic HIV infection or hypertension). And, people may opt not to use services because of perceived low quality, or preferred use of other sources of treatment (eg, traditional providers).
Likelihood of services	Proportion who visit a health facility or provider that is ‘ready’ (ie, all necessary inputs are available) to deliver the required services among those in need. This is also referred to as as input-adjusted coverage.	Service readiness or inputs: services cannot be provided as recommended if essential inputs are unavailable and inadequate (eg, facilities are not adequately stocked with essential medicines and equipment or basic running water and electricity, there are not enough trained health workers, etc).
Crude coverage	Proportion of the target population who receive a needed health intervention.	A health service is visited and all needed inputs are available for delivering the relevant intervention, but the intervention is not given. This may refer to the condition for which the individual sought health services, but may also be other opportunities related to, for instance, child vaccination.
Quality-adjusted coverage	Proportion of the target population receiving the service according to recommended standards (provider adherence to standards).	Interventions can only result in the intended health benefit if they are delivered in a respectful, timely fashion and according to standards and recommended guidance. Providers can also harm patients through the prescription of incorrect treatment.
User-adherence-adjusted coverage	Proportion of the target population receiving the service according to recommended standards and adhering to the treatment guidelines.	Several interventions require adherence of the user home treatment (eg, ARV therapy, antibiotic therapy, family planning methods) to maximise the effectiveness of treatment.
Outcome-adjusted coverage	Proportion of the target population experiencing the health gains from the service. This is also referred to as effective coverage. The framework proposes to use effective coverage only for the outcome-based coverage. The other levels of coverage are quality-adjusted measures of coverage. According to WHO, quality of care is the extent to which healthcare services provided to individuals and patient populations improve desired health outcomes. This definition implies a causal association of care received and impact, and is consistent with outcome-based definition of effective coverage.	Treatments have variable levels of efficacy, which implies that even if all standards are followed, health gains will be less than 100%. This applies to vaccines, family planning methods, antibiotics, etc.

ARV, antiretroviral.

**Table 2:** Components of coverage, data collection methods and indicators for selected interventions along the continuum RMNCH+N

Intervention	Target population	Service contact	Likelihood of service	Crude coverage	Quality-adjusted coverage	User adherence adjusted	Outcome-adjusted coverage (effective coverage)
Data collection and analysis methods	Population survey, surveillance, estimates from population projections	Population survey (recall), facility reports	Facility readiness assessment and population survey (linked analysis)	Population survey, facility reports	Population survey, facility reports; facility assessment with measurement of practices (linked analysis)	Follow-up surveys	Multiple indicators with facility data; surveys often with biomarkers
Interventions		Increase awareness population, improve access, community workers	Improve service supplies and training	Health worker training and supervision to reduce missed opportunities	Health worker training and supervision to improve standards	Education clients through public and individual channels; community follow-up	Research to improve efficacy prevention and treatment methods
Family planning	Sexually active women who do not intend to become pregnant	Woman visits health facility (for any reason)	Facility that is FP ready	Receives FP methods	Multiple methods choice; standards followed	Use modern methods according to protocol	No unintended pregnancy
Antenatal visit	Women who are pregnant	Visits ANC clinic	Facility that is ANC ready	Receives ANC interventions	All relevant interventions and according to standard	Use of selected interventions at home (eg, IFA)	Positive pregnancy outcomes
Delivery care	Women who are delivering	Deliver in a health facility	Facility that is delivery care ready	Receives delivery care (SBA, partograph, etc)	Receives all required delivery interventions according to standards	–	Perinatal and maternal health outcomes
Postnatal care	Women who have delivered; newborns	Visits PNC clinic	Facility that is PNC ready	Receives PNC interventions	Receives PNC interventions according to standard	Use of selected interventions at home	Newborn and maternal health/survival postpartum
Immunisation	Infants at different ages	Infant visits health facility	Facility that is immunisation ready	Receives vaccination	Receives vaccination according to standards	Timely vaccination according to age and standard	Seroconversion; incidence and mortality due to VPD
ORS treatment	Children with diarrhoea	Taken to health facility	Facility ready to provide ORS	ORS received (and other treatment/advice)	ORS received according to standards	Use of intervention at home	Mortality/nutrition consequences due to diarrhoea
ARI treatment	Children with suspected pneumonia	Taken to health facility	Facility ready to diagnose and provide treatment	Receives diagnosis and treatment	Treatment received according to standards	Use of intervention at home/adherence	Mortality and nutrition consequences due to pneumonia
HIV ART	Person living with HIV	Visits health facility	Facility ready to diagnose and provide treatment	Receives treatment	Receives treatment according to standards	Adherence to treatment	Viral load suppression and survival
Malaria treatment	Children with fever	Visits health facility	Facility ready to diagnose and provide treatment	Receives treatment	Receives treatment according to standards	Adherence to treatment	Mortality/nutrition consequences due to malaria
Nutrition	Household population; children; women	Visits health facility or outreach clinic	Facility ready to diagnose and provide treatment	Receives nutritional food	Receives nutritional food according to standards	Uses nutritional food according to standards	Malnutrition prevalence

ANC, antenatal care; ARI, acute respiratory infection; ART, antiretroviral therapy; FP, family planning; IFA, iron folic acid; ORS, oral rehydration therapy; PNC, postnatal care; RMNCH+N, reproductive, maternal, newborn, and child health and nutrition; SBA, skilled birth attendant; VPD, vaccine preventable disease.

## Synthesis of effective coverage literature

We reviewed the published literature since 2000 to support our framework for measuring dimensions of quality-adjusted or effective coverage. A total of 8103 publications on coverage of RMNCH+N since the year 2000 were obtained from PubMed and screened (see online supplementary appendix 1). In all, 36 papers were selected. To quantify the drop between contact coverage and quality-adjusted coverage, we retained the 32 papers that included both a measure of crude or contact coverage and a quality-adjusted coverage measure. We documented the methodological approaches applied and quantified the size of the gap between crude, quality-adjusted and effective coverage where possible.

10.1136/bmjgh-2018-001297.supp1 Supplementary data

**Figure 2:**
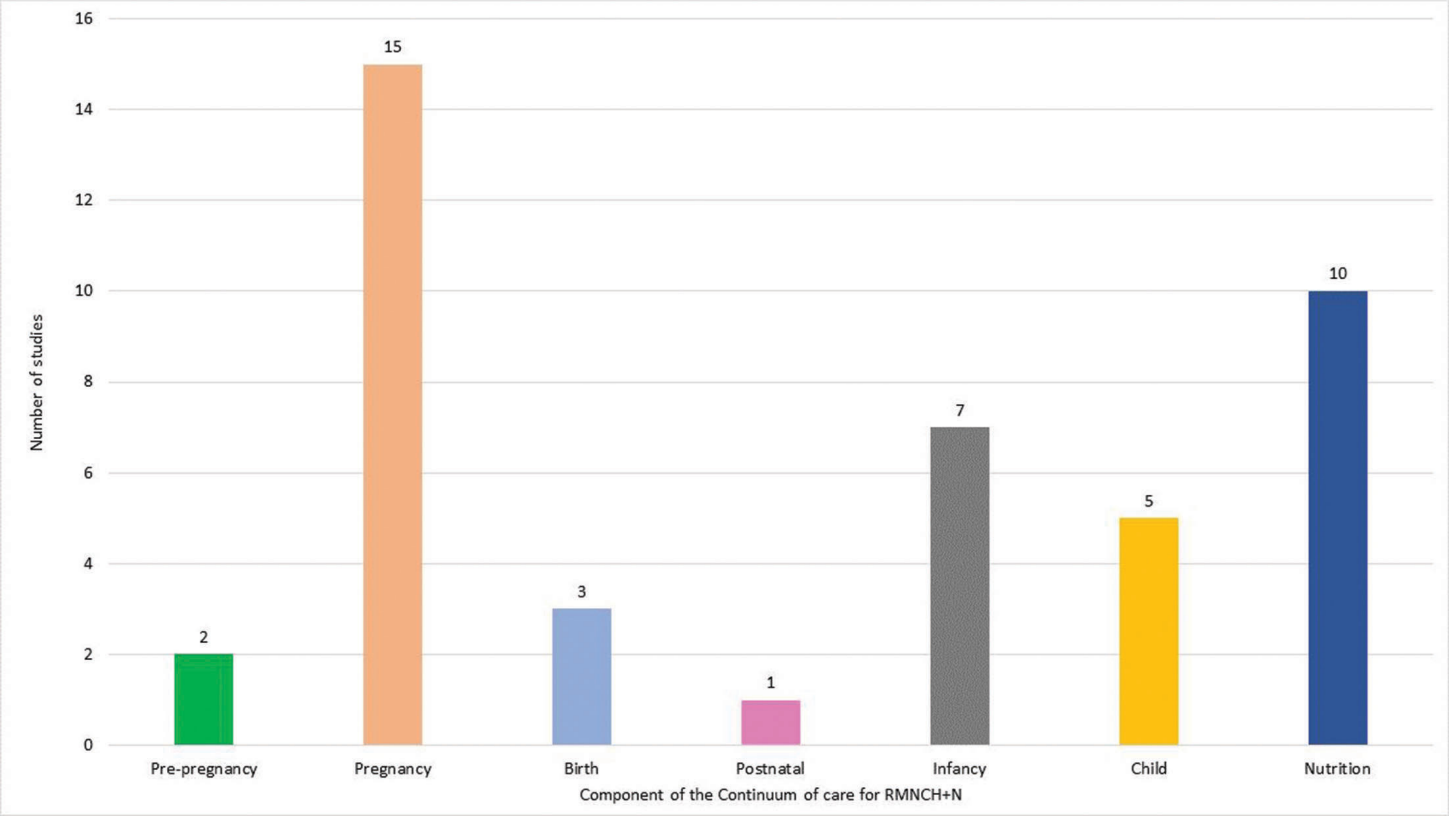
Distribution of publications by component of thecontinuum of care.

Of the 32 retained articles, 31 were carried out between 2010 and 2017, 22 since 2015. The bulk of the articles reviewed focused on antenatal care (ANC; 15 articles), nutrition (10 articles) and infancy (seven articles) (figure 2). Studies assessing coverage indicators for infant health dealt exclusively with immunisation while those for child health were either about treatment of child illness or use of bednets. Coverage of pre-pregnancy, birth and postnatal care interventions were the least documented. See online supplementary appendix 3 that includes full details of publications, interventions analysed and their target population and service contact, crude and quality-adjusted measures produced. Using our framework, we observed the following in different intervention areas:

- Few studies reported crude and adjusted coverage measures for interventions for the pre-pregnancy and birth periods. For pre-pregnancy, one study relied on use of modern contraceptives among women aged 15–49 years as crude measure and linked with a facility survey input measure to adjust for quality.^12^ The other used a demand satisfied with modern contraception as crude measure and adjusted with adherence to standards of care based on direct observation at facilities.^2^ The two studies that reported on births relied on linking between household survey and facility or frontline worker surveys. Both used skilled birth attendant as crude coverage measure and adjusted with facility input measures to estimate the likelihood of service coverage.^3 12^ The only study that reported crude and quality-adjusted measures for postnatal care used recall-based information from household surveys to estimate quality-adjusted coverage measures based on single or a combination of postnatal care interventions received.^3^
- Most analyses of ANC have relied on women’s recall of number of ANC visits and selected interventions received to measure service contact and crude coverage or quality-adjusted coverage and the gap between these two measures.^1–4 13–21^ Other studies have linked household surveys with facility surveys. These allow measurement of the drop between crude coverage and the likelihood of service, and quality-adjusted coverage using data on observations of clinical care.^1 2 20 21^
- For immunisation during infancy, crude coverage measures were based on vaccination information from recall or cards from household survey. Quality-adjusted coverage included serological tests to detect specific vaccine-related antibodies.^22–26^ One study adjusted the recall/card-based immunisation coverage measure with facility-level inputs to estimate the quality measure and another study considered timeliness and card availability.^12 27^
- Five studies analysed childhood interventions focusing on care seeking and treatment for child illness such as diarrhoea, fever and symptoms of acute respiratory infection, and use of insecticide-treated bednets. In addition to careseeking coverage, three of these studies measured process-adjusted coverage from recall of procedures and treatment received.^12 28 29^ Two studies linked household and facility surveys to measure input-adjusted coverage or process-adjusted coverage.^2 12^ One study carried out blood testing for *Plasmodium* *falciparum* to compare with the use of long-lasting insecticide-treated bednets.^30^
- Nutrition publications were mostly small-scale studies relying predominantly on recall of food consumption by women and children.^10 31–36^ Crude coverage was measured through consumption of/exposure to a particular fortified food; quality-adjusted measures were based on regular consumption of the fortified food, a user-adjusted coverage measure. One study reported on breast feeding among children under 6 months and another on home fortification with micronutrient powder among children 6–59 months.^12 37^

## Methods for measuring effective coverage in RMNCH+N

This review of effective coverage analyses showed considerable variability in study methods, including data sources, indicator definitions and analytical approaches, and not always consistent with the logical flow of our proposed framework (see online supplementary appendix 2). The greatest consistency was the source of data for defining intervention target population: one analysis employed a population cohort from a demographic surveillance site, whereas all others used cross-sectional household surveys, either programme specific or standard surveys such as Demographic and Health Survey (DHS) or Multiple Indicator Cluster Survey (MICS). Definitions of target population varied subtly; for instance, the recall period for women in need of ANC varied from currently pregnant to live birth in the past 5 years.^17 35^ Eligibility of children for effective coverage of child health services depended on the intervention and was sometimes based on age alone (eg, vaccination) or age as well as illness; some studies of nutrition limited the definition of those in need to children in poverty with poor diet diversity or suboptimal feeding practices.^32 34^ In measuring intervention coverage, the majority of studies relied on the same data source as in defining the target population and elicited self-reports of healthcare use (care seeking) or health commodity use (ie, treatment, contraceptive, supplemented food product). The exception was use of geospatial information on households and facilities to estimate geographical access to health facilities within 5 km of recently pregnant women.^19^

The largest variation in methods was observed in estimates of quality-adjusted estimates. Three types of data sources were used: self-report via the same population-based survey used for defining crude coverage, assessment of specimen samples collected during the survey (eg, blood titre for antibody response, food specimen for micronutrient concentration) and a separate sample of health facilities or, in one case, food available at local markets. Indicators of quality differed across nearly every study and included binary indicators of receipt of a single service (positive antibody titres, self-reported health commodity use), binary indicators of multiple elements (facility with resources required for quality, visit with most/all essential elements of evidence-based care included), and, least commonly, proportions of care available or delivered (adherence to evidence-based guidelines, proportion of necessary resources available in facility). Analytic approaches differed for studies linking individuals surveyed in households to external information such as health facility assessments: three studies linked individuals to facilities directly using health records,^20^ linear distance^19^ or cluster boundaries.^7^ All others relied on ecological linkages between summaries of access to care and quality of care stratified by region and/or facility characteristics, with little consistency in choice of strata. Calculation of variance around quality-adjusted coverage estimates was similarly heterogeneous. Studies relying on a single population survey for all indicators typically calculated variance following survey sample methods. Those combining sources most often reported no variance for effective coverage calculation. One study employed a Taylor series expansion^12^ and one the exact variance of a product.^2^

## Gap between crude coverage and quality-adjusted coverage

**Figure 3:**
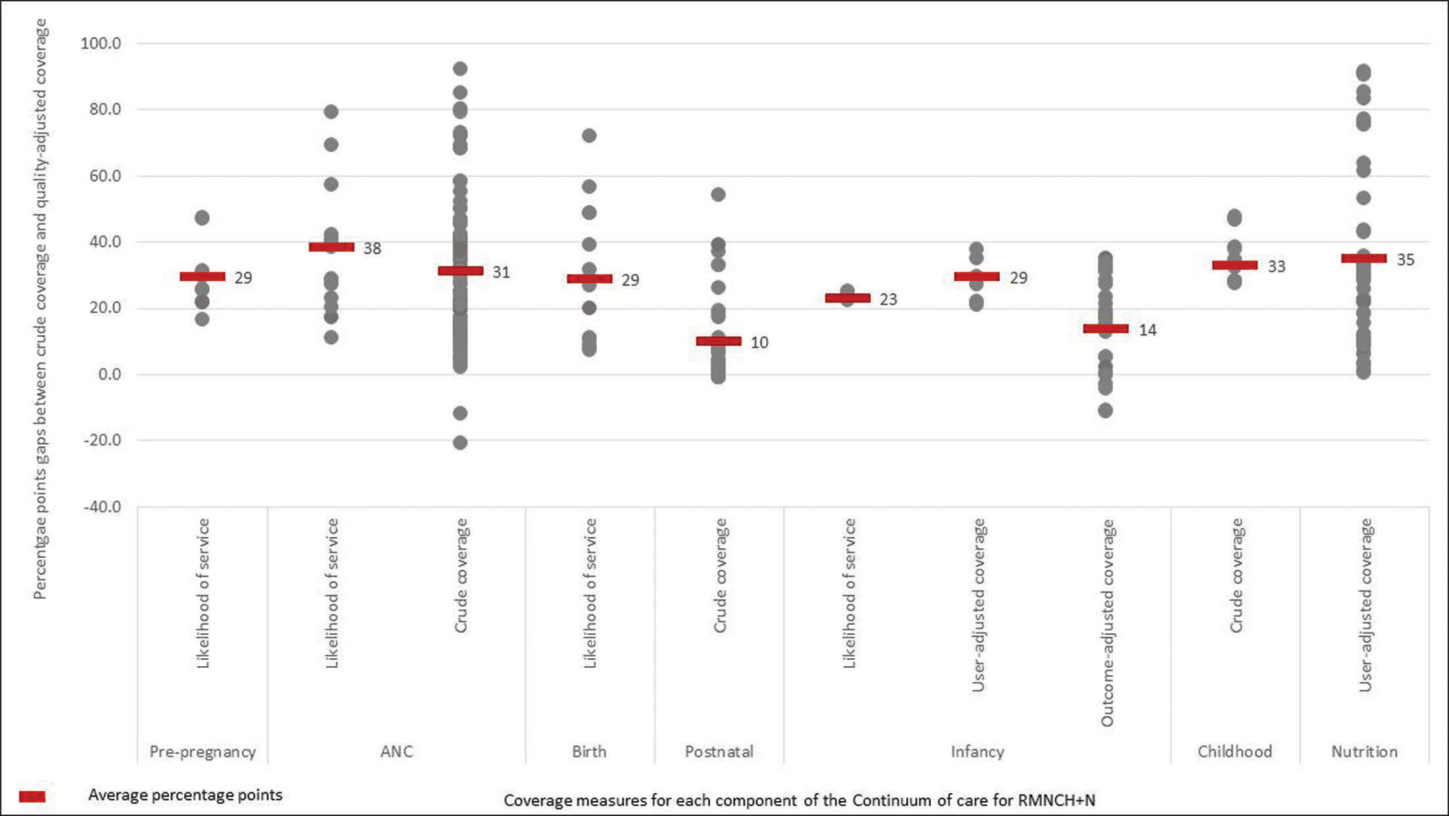
Average percentage points gap between contact or crudecoverage and adjusted coverage measures.

Figure 3 shows the average percentage point gaps between contact or crude measures and adjusted measures along the continuum of care. Studies have mostly measured two or three steps of the cascade, usually a contact indicator followed by an input-adjusted indicator or the likelihood of care, or crude coverage indicator and a quality-adjusted measure. Only studies assessing effective coverage of immunisation of infants included measures of outcome-adjusted coverage, measured using serological tests, independent from the crude coverage measure. Figure 3 demonstrates the evidence of a large drop in coverage when some measure of quality is used, ranging from an average percentage point drop of 10 to 38 points. The evidence suggests large variability in the size of the drop across the continuum of care. It also depends on the type of adjustment made, the indicators used and whether the baseline indicator is a contact indicator (eg, care seeking for treatment of child illness) or a crude coverage (measles vaccination). For ANC and nutrition, there were measures for which the gaps between contact or crude coverage and the quality-adjusted coverage were over 90 percentage points but also some measures which show higher quality-adjusted coverage than crude coverage. This is the case for immunisation of infants where serological tests resulted in higher coverage than the crude recall-based coverage. This may be the result of a measurement artefact as for example, in cases which compared ANC4+ as a crude coverage measure to coverage of receipt of one or a set of interventions during ANC as a quality-adjusted measure. Coverage of receipt of an intervention during ANC as measured through women’s recall may be higher than ANC4+.

## Key challenges measuring effective coverage

In the absence of a standard organising framework to guide the measurement of coverage indicators that adjust for quality dimensions, researchers have developed their own methods and adjustment approaches, focusing on specific components of quality of care, often relying on WHO-recommended standards for care provision.^2^ Building on previous attempts at developing a framework for effective coverage, such as the Tanahashi’s model, we propose a framework for measuring effective coverage in RMNCH+N that presents a cascade of potential loss of effectiveness of an intervention from contact with a health provider to effective coverage.^11^ We considered effective coverage as outcome-based coverage: proportion of individuals experiencing optimal health gains from a service among those who need the service. Our proposed framework contributes to harmonising the various definitions and terminologies of effective coverage currently used inconsistently in the scientific literature while maintaining the focus on impact in RMNCH+N.

Advancement in coverage measurement faces numerous challenges, reflected in the studies reviewed. Although these studies generally show a substantial drop in coverage when comparing contact or crude coverage to quality-adjusted measures, definitions, methods and approaches used are heterogeneous and inconsistent. Quality of care is a multidimensional construct that includes inputs, processes and outcomes and experience of care from the patient perspective. Due to lack of consensus on measurement, the operational definition used to assess measures that adjust for quality mostly depends on available data and study objectives. There are currently few standards in items or procedures, in terms of their composition and number, included in quality adjustment, whether for inputs, processes or outcomes. Studies have considered either single interventions and/or a combination of procedures using simple arithmetic averages. Thus, measures that adjust for quality, and therefore the gap between crude coverage and effective coverage, do not compare from one study to another. Similarly, for studies that have combined household and facility survey data to derive adjusted measures, there are no standard approaches for linking these datasets. Some studies have used geocoordinates for an ecological linkage, whereas others have used the type of facility and/or administrative area.^1 2^ In addition, there is substantial variation in the temporal gaps between household and facility assessments, in the sampling design of the facility assessment, and in the weighting of facility data for linked analyses.

Current studies have in most cases been opportunistic, often relying on secondary data from household interviews and facility surveys. Studies that have used primary standalone data collection were often of small scale, covering few districts. Consequently, each stage of the continuum of care for RMNCH+N is not equally covered, nor are all steps of the cascade framework captured. The area most covered is ANC due to widely available data on ANC content from household and facility surveys. No study assessed whether adjusting for dimensions of quality was associated with impact or with loss of effectiveness or impact.

The proposed cascade framework offers an organising approach for improving consistency and definitions across studies attempting to measure quality-adjusted coverage, as well as the interpretation on these measures. Although the cascade approach is intuitive for most interventions or packages of interventions, there may be situations where the coverage measures do not decrease monotonically along the steps. This is mainly the case for user-adjusted adherence, which can occur even though services were not delivered according to standard. In such cases, independent serial cross-sectional coverage measures at each step may not decrease along the steps. Such issue will, however, disappear when the coverage measure of each step effectively depends on the realisation of previous steps.

## Way forward for effective coverage measurement

Biomarkers and cohort registration approaches are central in the cascade frameworks used in programmes for the prevention of mother to child HIV transmission, HIV antiretroviral treatment and tuberculosis diagnosis and treatment.^38–42^ Biomarkers are used to assess the population in need and to measure outcomes of interest such as HIV viral load suppression. In immunisation, disease incidence is the main outcome interest, but seroconversion rates are used as a measure of a biological outcome of services and thus measure effective coverage.^25^ In maternal and newborn health programmes, biomarkers and cohort approaches are less common and effective coverage is often defined in terms of quality-adjusted coverage measures, based on the contents of services and the extent to which services were delivered according to standards. Cohort approaches in the context of maternity care can provide outcome data related to service provision. In other cases, new approaches that include multiple indicators and analytical methods to assess the health gains such as combining population survey data with health facility data including health outcomes such as institutional perinatal mortality rates should be tested.

Three critical recommendations stem out of our review. First, terminologies and definitions used for monitoring effective coverage must be standardised and harmonised across the RMNCH+N at global level and within the scientific community. Only then will the coverage cascade and effective coverage measures be more easily interpreted, meta-analysed and communicated to countries resulting in policy and programmatic action. Our proposed framework, which is sufficiently broad and applicable to all components of the RMNCH continuum, offers such standardisation.

Second, measuring each step on the framework will require improvements in data collection at both household and facility levels, as well as investment in stable national and subnational surveillance systems. Measuring populations in need from household surveys will require innovations in data collection, including for example biomarkers. Data sources for estimating coverage at national, regional and global levels have relied mainly on national household surveys, driven primarily by the DHSs initiated in the mid-1980s and the MICSs implemented since mid-1990s.^43 44^ Although the size of the questionnaires used by these programme has grown substantially over time, limited progress has been made in indicator development for RMNCH+N coverage, and most indicators have been measured in the same way for the past 20–30 years.^45^ Furthermore, more rigorous validation of household survey-based RMNCH+N content indicators using either observation of service delivery and follow-up recall interviews with women or biomarkers in some cases has uncovered inaccuracy in some key indicators.^46–49^ Recall-based household interviews do not measure correctly most interventions delivered during intrapartum care.^49^ Similarly, some indicators of treatment for childhood illnesses measured in household surveys (antibiotics for symptoms of pneumonia, antimalarial for fever) have been proven invalid for monitoring coverage of these treatment interventions.^50–52^ Improved measurement of service contact, likelihood of service and quality-adjusted coverage measures will also require investment in improving facility data, both routine health system information and standalone health facility surveys, developing best approaches for collecting linked data. There are also major gaps in measuring user adherence and client experience services, which will require special innovative approaches.

Third, approaches that link household-based data with health facility data are increasingly popular, but priority must also be given to analytical techniques for the linking and computation of valid measures of effective coverage.^53^ The few studies that have implemented such linking have used ecological linking and relied on existing data such as DHS and Service Provision Assessment (SPA) or Service Availability and Readiness Assessment (SARA) surveys to estimate quality-adjusted coverage measures.^1 2^ Those that have used primary data were at small scale.^3 54^ The linking was implemented using geocoordinate data or facility type disaggregated by urban/rural location. Furthermore, variance and precision of coverage indicators derived from linked data are yet to be fully understood.

While the evidence based on methods for effective coverage measurement must be expanded, it is critical that the global community, including national data collection programmes such as DHS, MICS, SARA, SPA, prioritises reporting of each step of the proposed cascade framework in RMNCH+N. Measurement to better monitor, understand and act on the gaps in effective coverage is required to make significant progress towards universal health coverage with quality services for women’s and children’s health.
