# Supplementary material for: Advances in the measurement of coverage for RMNCH and nutrition: from contact to effective coverage
Source: BMJ Glob Health. 2019 Jun 24;4(Suppl 4):e001297. doi: 10.1136/bmjgh-2018-001297 (PMC6590972; doi:10.1136/bmjgh-2018-001297)
Supplement: Supplementary data [file bmjgh-2018-001297supp001.pdf]

Appendix 1

Comprehensive literature search on effective coverage for RMNCH & N interventions

We carried out a comprehensive search of published literature using the PubMed database to identify publications which included measures of crude or contact coverage and at least one measure of quality-adjusted indicators (as depicted on figure 1). The search was conducted on interventions along the continuum of care including reproductive health or family planning, antenatal care, delivery care, and postnatal care, infant and young child nutrition, immunization, and treatment of childhood illnesses (malaria, diarrhea and pneumonia). We did not include environmental health interventions such as water and sanitation, and we focused on low and middle income countries. To prioritize recent evidence, only articles published between 2000 and October 2017 were retained. The search was carried out iteratively, starting with the most salient search terms related to effective coverage or quality-adjusted measures (table 1). Terms identified from the key words from the articles retrieved from the first search were then added (table 2). The search was carried out using EndNote Software version 7. Two additional references that were published after the search was completed were identified through known experts in the field of quality of care and effective coverage for a total of 8103 articles identified. To screen the references identified, inclusion and exclusion criteria were defined and applied (Box 1). Two authors (AA, MR) carried out the screening. In the case of disagreement, the two authors reviewed the abstracts or the full articles together to resolve the disagreement. Conference calls were also held with a group of co-authors to review progress. Figure 2 shows the steps involved in the screening process. The review process consisted in screening first the title of the references and in some cases, abstracts to identify potentially relevant papers based on the inclusion/exclusion criteria. Inclusion and exclusion criteria were used to eliminate publications that were evidently not relevant as suggested by their title. This resulted in 290 papers being identified as potentially relevant to effective coverage measurement. The next step involved the review of abstracts only. Only papers that described results indicating potential measures of contact and quality-adjusted coverage measures were retained based on the inclusion/exclusion criteria. Quality-adjusted coverage measures are generally refinement of contact indicators to account for some measure of quality of care. This resulted in 62 papers being selected. The final stage involved downloading full publications for review. Papers that included measures of quality-adjusted coverage were abstracted to provide a summary of method used. A total of 36 papers were abstracted. To quantify the drop between contact coverage and quality-adjusted we retained papers that included measure of contact indicators and at least a quality-adjusted coverage measure. Two papers that did report this information were not included in this stage.

Table 1: Initial search terms used in the literature search of effective coverage

RMNCH and Nutrition intervention	Search terms
Family planning/reproductive health	Effective coverage; coverage and quality of care; contact and content interventions; effective coverage and bottleneck of implementation; coverage and missed opportunities; coverage and adequate care; linking household survey and health facility; linking household and service provisional assessment; immunization and seroconversion rate
Antenatal / pregnancy care	
Delivery care	
Immunization	
Treatment of sick children	
Nutrition coverage	

Box 1: Inclusion and Exclusion criteria for the literature search

Inclusion criteria

1. Studies carried out since 2000
2. Studies using primary or secondary population or facility-based data to estimate contact or crude and "quality-adjusted" coverage focusing on at least one of the coverage measures above
3. Studies using primary or secondary population or facility-based data to carry out linkage between household data and facility level data to measure coverage
4. Studies measuring direct health gains due specific RMNCH& Nutrition interventions with established causal link between intervention and health gain

Exclusion criteria

1. Studies carried out before 2000
2. Studies not focusing on RMNCH & Nutrition intervention areas listed above
3. Studies estimating crude coverage without further attempt to adjust for quality of care
4. Studies that do not measure coverage with an effort to measure the population in need of the intervention (e.g. studies exclusively on program users or facility users)
5. Reviews, policy and viewpoint papers with no quantitative analysis and results
6. Studies in developed countries

Figure 2: Literature search process and screening of reference.

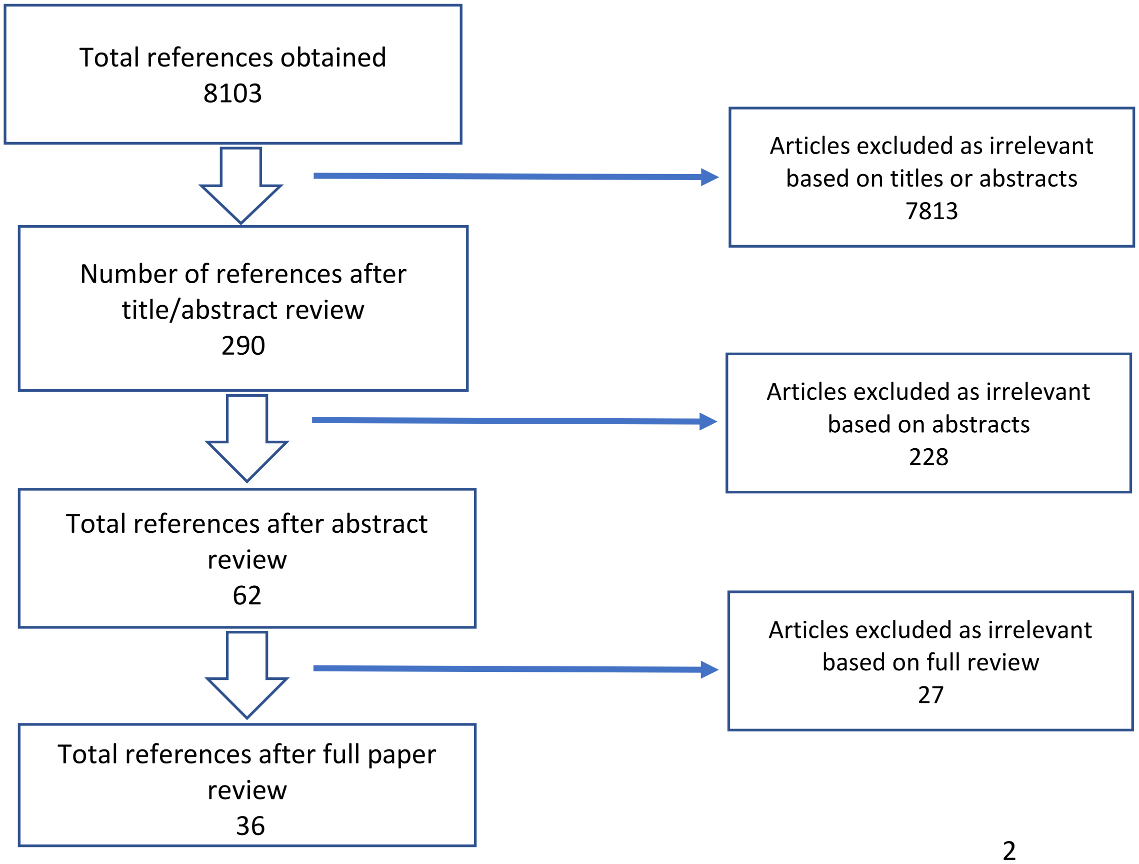

Table 2: Second stage search terms (merged with the initial search)

Search terms	Numbers reference hit
1. Coverage AND (ANC OR “antenatal care” OR “pregnancy care”)	1345
2. Coverage AND (“postnatal care OR “postnatal home visits” OR “newborn care”)	621
3. Coverage AND (breastfeeding OR “infant feeding”)	234
4. Coverage AND (“facility-based delivery” OR “institutional delivery” OR “home delivery” OR “facility births” OR “home births”)	26
5. Coverage AND (“MCH Interventions” OR “maternal and child health interventions”)	724
6. (Coverage AND “content interventions”)	1249
7. Coverage AND (“childhood immunizations” OR “childhood vaccinations”)	231
8. (Coverage AND “missed opportunities”)	110
9. (Coverage AND “malaria prevention” AND “pregnant women”) OR (Coverage AND “malaria prevention” AND “children”)	337
10. (Coverage AND “malaria treatment” AND “pregnant women”) OR (Coverage AND “malaria treatment” AND “children”)	36
11. (Coverage AND “nutrition interventions”)	63
12. Coverage AND (“health service delivery” OR “health service provision”)	60
13. Coverage AND (“Community Health Workers” OR “CHW”)	74
14. (Coverage AND “care seeking” AND “childhood illness”) OR (Coverage AND “care seeking” AND “newborn illness”) OR (Coverage AND “care seeking” AND “pneumonia”) OR (Coverage AND “care seeking” AND “diarrhea”)	6
15. (Coverage AND “high quality contact” AND “health worker)	0
16. (Coverage and “facility readiness”)	5
17. (“Gaps in coverage” AND “bottleneck”) OR (coverage AND bottleneck AND intervention”)	53
18. (Coverage AND utilization AND “Access to care”)	1653
19. (“availability coverage” AND “health facility” and “availability coverage”)	146
20. (“accessibility coverage” AND “health facility” and “accessibility coverage”)	310
21. (Coverage AND “family planning” AND “services”)	134
22. (Coverage AND “HIV”)	507
TOTAL hit	**8099**

APPENDIX 2 - SYNTHESIS

PRE-PREGNANCY (Reproductive health/family planning)

Reference	Intervention	Target population	Data	Country (geographical scope)	Service contact (utilization)	Likelihood of service	Crude coverage	Quality-adjusted coverage
Nguhiu PK et al. 2017.	Family planning services	Women 15–49 years old who at the time of survey were able to get pregnant (fecund)	Kenya DHS 2003, 2008, 2014 and Kenya SPA	Kenya			% Fecund women 15–49 years old, currently using a modern contraceptive method	CPRm adjusted by facility level score based on the presence of client privacy during consultation, availability of reproductive health counselling visual aids and record tools, and reproductive health commodity management practices in a facility. (source KSPA linked with KDHS)
				2014			67.80%	40.70%
				2008/2009			62%	36.60%
				2003			53.70%	31.90%
Leslie HH et al . 2017	Family planning services	women 15-49 years married or in union who wish to space or limit childbearing	DHS/MICS surveys linked with SPA in the past decade (with observation)	8 countries: Haiti, Kenya, Malawi, Namibia, Rwanda, Senegal, Tanzania, uganda,			Demand satisfied for modern contraception	Effective coverage: product of crude coverage and quality score of family planning based on percent of 16 items*
				Haiti			46.7% ±1.1%	16.7%±0.5
				Kenya			57.9%±1.3%	27.3%±0.9
				Malawi			77.6%±0.6%	30.3%±1.%
				Namibia			84.2%±0.7%	37.3%±1.1%
				Rwanda			63.4%±0.8%	37.9%±1.2%
				Senegal			40.9%±1.5%	19.3%±1.2
				Tanzania			56.5%±1.1%	25.3%±0.7%
				Ugandan			34.9%±1.4%	18.4%±1.6%

* the 16 items include: reproductive history (age, living children, last delivery date, pregnancy complications, last menstrual period, desire for child/more children, desired timing for birth or next child, breastfeeding, menses), Health history/exam (blood pressure, weight, smoking, STI symptoms, chronic illness, pelvic exam), counsel on method (any counseling on method)

Note: There were no data related to user-adjusted or outcome-adjusted coverage

Antenatal Care

Reference	Intervention	Target population	Data	Country (geographical scope)	Service contact (utilization)	Likelihood of service	Crude coverage	Quality-adjusted coverage	user-adjusted coverage	Outcome-adjusted coverage
Baker et al. 2015a.	Syphilis screening during ANC	Women with a live birth in previous 12 months	Household and facility surveys	Tanzania	ANC (≥1 vist)	ANC in a facility with syphilis test availbale	ANC in a facilty with syphilis test and receipt of blood test and test results for syphilis			
				Tandahimba district	99%	47%	12%			
				Newala district	100%	77%	20%			
Hodgins et al, 2014.		Women with a live birth in previous 24 months	DHS data in 41 countries	41 countries with DHS data available	ANC (≥4 visits)		Receipt of blood pressure test during ANC visit			
	Blood pressure screening during ANC				57%		78%			
	Tetanus (2 doses)				57%		69%			
	Iron-folic acid supplementation for 90 days				57%		21%			
	Counseling on pregnancy danger signs				57%		49%			
	Urine sample testing				57%		55%			
	HIV counseling and testing				57%		43%			
	IPTP (2 doses of SP)				57%		20%			

Antenatal Care

Reference	Intervention	Target population	Data	Country (geographical scope)	Service contact (utilization)	Likelihood of service	Crude coverage	Quality-adjusted coverage	user-adjusted coverage	Outcome-adjusted coverage
Marchant T et al. 2015.	Set of eight ANC processes (Weight and height measured; blood pressure measured; urine and blood tests carried out; counselling for breastfeeding, danger signs, and birth preparedness)	Women with a live birth in previous 12 months	Household surveys and frontline worker and facility surveys in selection areas in each country		ANC (≥1 vist)		Women who had at least one antenatal care visit and for whom all eight antenatal processes were met			
				Nigeria (Gombe State)	61% (50-72)		11%			
				Ethiopia (Oromia, Tigray, Amhara and SNNP)	56% (49-63)		4%			
				India (Uttar Pradesh)	74% (69-79)		6%			
							weight measured			
				Nigeria (Gombe State)	61% (50-72)		56% (45-66)			
				Ethiopia (Oromia, Tigray, Amhara and SNNP)	56% (49-63)		45 (40–54)			
				India (Uttar Pradesh)	74% (69-79)		32 (27–36)			
							Height measured			
				Nigeria (Gombe State)	61% (50-72)		35 (26–44)			
				Ethiopia (Oromia, Tigray, Amhara and SNNP)	56% (49-63)		27 (22–32)			
							Blood pressure measured			
				Nigeria (Gombe State)	61% (50-72)		57 (47–68)			
				Ethiopia (Oromia, Tigray, Amhara and SNNP)	56% (49-63)		41 (36–47)			
				India (Uttar Pradesh)	74% (69-79)		34 (29–39)			
							Urine tested			
				Nigeria (Gombe State)	61% (50-72)		36 (26–48)			
				Ethiopia (Oromia, Tigray, Amhara and SNNP)	56% (49-63)		24 (19–30)			
				India (Uttar Pradesh)	74% (69-79)		33 (28–38)			
							Blood tested			
				Nigeria (Gombe State)	61% (50-72)		45 (35–55)			
				Ethiopia (Oromia, Tigray, Amhara and SNNP)	56% (49-63)		47 (40–54)			
				India (Uttar Pradesh)	74% (69-79)		24 (20–29)			
							Counselled about breastfeeding			
				Nigeria (Gombe State)	61% (50-72)		49 (39–60)			
				Ethiopia (Oromia, Tigray, Amhara and SNNP)	56% (49-63)		34 (28–40)			
				India (Uttar Pradesh)	74% (69-79)		28 (23–32)			
							Counselled about danger signs			
				Nigeria (Gombe State)	61% (50-72)		41 (31–51)			
				Ethiopia (Oromia, Tigray, Amhara and SNNP)	56% (49-63)		24 (19–29)			
				India (Uttar Pradesh)	74% (69-79)		28 (23–32)			
							Counselled about birth preparedness			
				Nigeria (Gombe State)	61% (50-72)		46 (35–57)			
				Ethiopia (Oromia, Tigray, Amhara and SNNP)	56% (49-63)		36 (30–43)			
				India (Uttar Pradesh)	74% (69-79)		29 (24–34)			

Antenatal Care

Reference	Intervention	Target population	Data	Country (geographical scope)	Service contact (utilization)	Likelihood of service	Crude coverage	Quality-adjusted coverage	user-adjusted coverage	Outcome-adjusted coverage
Thapa et al. 2016	Calcium supplementation during pregnancy (this was an operation research)	Women who delivered in the past six months	Household survey of postpartum women who delivered in the past 6 months	1 district in Nepal	ANC>=1		Women provided with calcium during ANC		Full compliance (consumed full course of the calcium)	
					94.60%		94.60%		67.30%	
Ndyomugyenyi R et al. 2010.	Intermittent Preventive Treatment of malaria in Pregnancy.	Women who had given birth within one year prior to the survey	Community interviews (unclear how sample was selected)	1 district in Uganda	ANC>=1		Received at least two doses of SP			
					94%		52%			
Kanyangarara et al. 2017.	(i) tetanus toxoid vaccine for pregnant women; (ii) intermittent preventive treatment of malaria in pregnancy (IPTp); (iii) syphilis detection and treatment in pregnancy; (iv) hypertensive disease case management, including management of pre–eclampsia with magnesium sulphate; and (v) iron supplementation	Women who delivered in the three years preceding the survey	DHS surveys and SPA/SARA surveys in 13 countries (17 DHSs and 20 SPA/SARA)	13 countries, all in sub-Saharan Africa	ANC1+ (median across 17 DHS surveys)		% women with blood sample taken (median) [recalculated to include all women in the denominator			
					93.10%		77%			
							% women with urine sample taken (median)			
					93.10%		62%			
							% women with weight measured (median)			
					93.10%		88%			
							% women who were given iron tablets or syrup (median)			
					93.10%		74%			
							% women with any or complete tetanus protection at birth (median)			
					93.10%		76%			
							% women who took any SP/Fansidar during visit (median)			
					93.10%		38%			
					ANC4+ (median across the 17 surveys)	% women with high likelihood of appropriate care (high likelihood of appropriate care if they attended a health facility type that in the SPA/ SARA had the necessary equipment, diagnostics, medicines and commodities in stock, was equipped with ANC guidelines, and had at least one staff member who had been trained recently;				
						Tetanus toxine vaccine for pregnant women (mean across 20 surveys0				
					49.20%	21.30%				
						IPTp				
					49.20%	20.40%				
						Syphilis detection and treatment				
					49.20%	8.40%				
						Hypertensive disease mangement				
					49.20%	9.40%				
						Iron supplementation				

Antenatal Care

Reference	Intervention	Target population	Data	Country (geographical scope)	Service contact (utilization)	Likelihood of service	Crude coverage	Quality-adjusted coverage	user-adjusted coverage	Outcome-adjusted coverage
					49.20%	22.30%				

Antenatal Care

Reference	Intervention	Target population	Data	Country (geographical scope)	Service contact (utilization)	Likelihood of service	Crude coverage	Quality-adjusted coverage	user-adjusted coverage	Outcome-adjusted coverage
Heredia-Pi, et al2016.	Antenatal care with skilled personnel (nurse of physician); timely ANC (ANC in the first trimester of pregnancy), sufficient ANC (at least 4 ANC visits), appropriate in content (summary of 8 procedures: weight, height, blood pressure, urine analysis, blood analysis, tetanus, vaccination, prescription of folic acid, prescription of vitamin iron or dietary supplements)	Women with a live birth during last pregnancy in the seven years preceeding the survey (from 2006 to 2012)	Data from the Mexican National Health and Nutrition Survey (ENSANUT) in 2012	Mexico	ANC1+ with skilled personnel		Appropriate ANC (7 out of 8 procedures received)			
					98.4 (98.1 to 98.8)		84.7 (83.3 to 86.2)			
					ANC4+ (Sufficient care)					
					91.4% (90.3 to 92.5)					
					Timely care (initial antenatal care visit during the first trimester of pregnancy)		% women with adequate ANC (timely + sufficient + appropriate)			
					83.2% (81.8-o 84.6)		71.5% (69.7 - 73.2)			
Kyei NN et al. 2012.	Folate/Iron Supplementation given or bought; Tetanus vaccination received; VCT for HIV offered; IPTof malaria taken, drug for intestinal parasites taken, weighed, height measured; blood pressure taken; blood sample given; urine sample given; urine sample given	4148 births (counting multiple pregnancies as one birth) in women 15-59 yrs between 2002 and 2007	Analysis of 2 national datasets with detailed antenatal provider and user information, the 2005 Zambia Health Facility Census and the 2007 Zambia Demographic and Health Survey (DHS), to describe the level of ANC service provision at 1,299 antenatal facilities in 2005 and the quality of ANC received by 4,148 mothers between 2002 and 2007.	Zambia	% women reported at least one ANC visit with a skilled health worker		% of mothers received at least moderate quality ANC (defined as 4+ ANC with skilled HW and receipt of at least 5 interventions			
					94%		53%			
					% women reported they received 4 ANC with Skilled Health Worker		% of mothers received good quality ANC (defined as 4+ANC with skilled HW and receipt of 8 interventions)			
					58%		29%			
							Folate/iron given or bought			
							91%			
							Tetanus vaccination received			
							79%			
							VCT for HIV offered			
							53%			
							IPT of malaria taken			
							87%			
							Drug for Inestinal parasites taken			
							36%			
							Weighed			
							89%			
							Height measured			
							26%			
							Blood pressure taken			
							81%			
							Blood sample given			
							59%			
							Urine Sample Given			
							22%			
							Birth preparedness discussed			
							72%			
Agha S et al. 2016.	Timing of first ANC; content of services ANC services in Sindh, Pakistan	women aged 15-49 with a live birth 2 years preceding the survey	2013 Representative household survey of women 15-49 with a live birth 2 years preceding survey in Sindh	Sindh province, Pakistan	ANC (≥1 vist)		Blood pressure measured			
					87%		70%			
							Blood test taken			
							62%			
							Urine test			
							63%			

Antenatal Care

Reference	Intervention	Target population	Data	Country (geographical scope)	Service contact (utilization)	Likelihood of service	Crude coverage	Quality-adjusted coverage	user-adjusted coverage	Outcome-adjusted coverage
							Took iron tablets			
							56%			
							2+ tetanus injection			
							53%			
							Weight measured			
							48%			

Antenatal Care

Reference	Intervention	Target population	Data	Country (geographical scope)	Service contact (utilization)	Likelihood of service	Crude coverage	Quality-adjusted coverage	user-adjusted coverage	Outcome-adjusted coverage
Baker et al. 2015b	bottlenecks in effective coverage of screening for Syphilis, HIV and anemia during ANC	women of reproductive age (13-49) with a live birth in past 12 months	household surveys of women of reproductive age, facility surveys and key informant interviews	Tanzania and Uganda	proportion attending ANC (≥1 vist for syphillis/HIV or ≥2 for anemia):	proportion attending ANC in a health facility where Syphilis/HIV/anemia tests are available	proportion who reported test result for Syphilis/HIV or who received blood test (anemia)			
				Tanzania (Newala)	Syphilis	Syphilis	Syphilis			
					100%	43%	15%			
					HIV	HIV	HIV			
					100%	83%	65%			
					Anemia	Anemia	Anemia			
					97%	55%	51%			
				Uganda (Namayingo)	Syphilis	Syphilis	Syphilis			
					95%	16%	3%			
					HIV	HIV	HIV			
					95%	56%	37%			
					Anemia	Anemia	Anemia			
					92%	23%	19%			
				Mayuge district, Uganda			Use of iron and folic acid during pregnancy: Proportion who took tablets or syrup for 30 or less days		Proportion who took tablets or syrup for 90 days.	
							99%		0%	
					Intermittent presumptive treatment of malaria: Proportion of women who attended ANC clinic during the pregnancy.			Proportion of women who received at least two doses of SP for malaria prophylaxis		
					92%			53%		
							HIV counseling during pregnancy: Proportion of women who got information related to HIV/AIDS and on being tested for the HIV virus.	Proportion of women who gave blood for testing during the pregnancy, who received HIV test results		
							76%	66%		
					Syphilis testing during pregnancy: Proportion of women who attended ANC clinic during the pregnancy			Proportion of women who gave blood for testing during the pregnancy, who received syphilis test results		
					92%			23%		
				Namayingo district, Uganda			Use of iron and folic acid during pregnancy			
							99%			
							Intermittent presumptive treatment of malaria	Proportion of women who received at least two doses of malaria prevention m		
							93%	55%		
							HIV counseling during pregnancy	Proportion of women who gave blood for testing during the pregnancy, who received HIV test results		
							68%	56%		

Antenatal Care

Reference	Intervention	Target population	Data	Country (geographical scope)	Service contact (utilization)	Likelihood of service	Crude coverage	Quality-adjusted coverage	user-adjusted coverage	Outcome-adjusted coverage
							Syphilis testing during pregnancy	Proportion of women who gave blood for testing during the pregnancy, who received syphilis test results		
							93%	14%		

Antenatal Care

Reference	Intervention	Target population	Data	Country (geographical scope)	Service contact (utilization)	Likelihood of service	Crude coverage	Quality-adjusted coverage	user-adjusted coverage	Outcome-adjusted coverage
Larsson EC et al. 2012.	HIV testing uptake during pregnancy	pregnant women	survey conducted May-July 2008	Iganga/Mayuge HDSS site in Eastern Uganda	Proportion of women who had at least 1 ANC	Coverage of HIV testing and counseling at facilities that have HIV testing services	proportion of women who were HIV tested during pregnancy			
					96%	85%	64%			
Nguhiu PK et al. 2017.	Functional ANC services	women 15-49 with at least one child under 5 years	Kenya DHS 2003, 2008, 2014 and Kenya SPA		Proportion with at least 4 ANC for last birth		ANC4 adjusted by individual-level score if respondent recalled any of the following performed during any ANC: BP taken, blood sample taken, urine sample taken, informed on pregnancy complications, iron tablets prescribed, drug for intestinal parasites prescribed			
				Kenya						
				2014	58.2 (56.5-59.9)		44.6 (43.2-46.0)			
				2008/2009	47.1 (44.7-49.5)		31.6 (29.8-33.4)			
				2003	52.3 (50.2-54.4)		31.7 (30.0-33.4)			
Deming MS et al. 2002.	Neonatal Tetanus	mothers of children < 1 year	MICS survey in Central African Republic 1996, follow-up of 1994-95 DHS sample. Collection of blood sample from mother of children < 1 year for testing for presence of tetanus antitoxin titres	Central African Republic	proportion of mothers who attended at least 1 ANC during last pregnancy;	proportion of mothers who were able to show vaccination card				
					77.90%	57.70%				
					proportion of mothers who delivered in a health facility/trained birth attendant		proportion of mothers protected at delivery according to history of TT vaccination			Tetanus antitoxin seroprevalence proportion among mothers tested
					63.50%		74.4% (67.0-81.7)			88.7% (83.2-94.2)
Leslie HH et al . 2017.	ANC services (At least 1 ANC visit, and 4+ visits)	women 15-49 years with a live birth in the two years preceding the survey	DHS/MICS surveys linked with SPA in the past decade	8 countries: Haiti, Kenya, Malawi, Namibia, Rwanda, Senegal, Tanzania, uganda,	ANC (4+ visit)			Effective coverage= product of crude coverage and quality score defined as Quality defined as the percent of the following 21 or 22 items performed. Items recommended once or twice during three follow-up visits are down-weighted by one third and two thirds respectively*		
				Haiti	64.8%±1.5%			29.1%±0.8%		
				Kenya	45.0%±1.6%			28.5%±1.1%		
				Malawi	46.2%±0.8%			23.2%±0.6%		
				Namibia	79.3%±1.2%			51.6%±1.5%		
				Rwanda	36.0%±1.1%			22.5%±0.8%		
				Senegal	45.8%±1.3%			26.2%±0.8%		
				Tanzania	48.1%±1.3%			21.2%±0.8%		
				Ugandan	45.2%±1.2%			24.3%±0.9%		

Antenatal Care

Reference	Intervention	Target population	Data	Country (geographical scope)	Service contact (utilization)	Likelihood of service	Crude coverage	Quality-adjusted coverage	user-adjusted coverage	Outcome-adjusted coverage

* item included are: History (Last menstrual period to calculate gestational age; Prior pregnancy experience, e.g. stillbirth, neonatal death, heavy bleeding, assisted delivery, multiples, and spontaneous or assisted abortion; Danger signs in current pregnancy, e.g. bleeding, fever, headache or blurred vision, swollen face or hands, tiredness or breathlessness, fetal movements; Previous complications on record) Previous complications on record); Routine Examination (Weight, fundal height, edema, vaginal exam, blood pressure, fetal heart rate, ultrasound) Sreening (syphilis test, HIV test, Anemia test, blood group test, urine test) Preventive measures (Prescribed or gave iron or folic acid or both, Provider prescribed or gave tetanus toxoid injection, Prescribed or gave intermittent preventive treatment in pregnancy (IPTp)) Education (nutrition, Sleeping under an insecticide-treated net, Delivery planning: preparation (money, transport) and location, Emergency planning: supplies for home delivery, Breastfeeding, Post-partum and postnatal care, Pregnancy spacing); Record keeping (Provider completed ANC card)

BIRTH

Reference	Intervention	Target population	Data	Country (geographical scope)	Service contact (utilization)	Likelihood of service
Marchant T et al. 2015.	Delivery care (Prevention of haemorrhage during SBA: administration of prophylactic uterotonics to prevent post-partum haemorrhage; active management of third stage of labour	Women with a live birth in previous 12 months	Household surveys and frontline worker and facility surveys in selection areas in each country		SAB%	Percent of women who were attended at birth by a skilled birth attendant and received active management of third stage of labour (AMTSL: Administration of prophylactic uterotonic, controlled cord traction, uterine massage)- Based on frontline health worker interview
				Nigeria (Gombe State)	22%	8%
				Ethiopia (Oromia, Tigray, Amhara and SNNP)	15%	4%
				India (Uttar Pradesh)	76%	4%
						Percent of women who were attended by a skilled birth attendant who administered a prophylactic uterotonics - Based on frontline health worker interview
				Nigeria (Gombe State)	22%	13%
				Ethiopia (Oromia, Tigray, Amhara and SNNP)	15%	7%
				India (Uttar Pradesh)	76%	37%
Nguhiu PK et al. 2017.	Skilled delivery and perinatal care	women 15-49 with at least one child under 5 years	Kenya DHS 2003, 2008, 2014 and Kenya SPA	Kenya	Proportion of women who reported a skilled delivery for last birth	SBA adjusted by facility-level score based on reported routinely performed essential newborn care practices at the facility maternity: routine rooming of the mother, routine weighing of the newborn, complete examination of newborn before discharge, administration of BCG before discharge, etc.
				2014	61.8 (60.3-63.3)	51.3 (49.8-52.8)
				2008/9	43.8 (40.5-47.1)	36.7 (35.0-38.4)
				2003	52.3 (50.2-54.4)	32.6 (30.8-34.4)
Larson E et al 2017.	Obstetritc and newborn care	Women with delivery in the 12 months prior to interview	Household survey, Health facility provider interview, facility audits	Tanzania (Pwani region)	Proportion of woment who delivered in a facility (adjusted to study facilities)	Five quality dimensions in cascade (Using minimum quality threshold of 50% of quality elements; Using a minimum of 90%, effective coverage was 0)
					81.60%	Infrastructure 55%
					81.60%	Infrastructure, Equipment, supplies, and drugs 50%
					81.60%	Infrastructure, Equipment, supplies, and drugs, provider knowledge and competence 33%
					81.60%	Infrastructure, Equipment, supplies, and drugs, provider knowledge and competence, routine obstetric care 33%
					81.60%	Infrastructure, Equipment, supplies, and drugs, provider knowledge and competence, routine obstetric care, basic Emergency Obstetric and newborn care 25%

POSTNATAL

Reference	Intervention	Target population	Data	Country (geographical scope)	Service contact (utilization)	Likelihood of service
Marchant T et al. 2015.	Post-partum care (Breasts and bleeding checked; counselled on danger signs, nutrition, and family plan)	Women with a live birth in previous 12 months	Household surveys and frontline worker and facility surveys in selection areas in each country		PNC Woman	the percent of women who had a post-partum check within 48 hours of birth and for whom all five post-partum processes were met
				Nigeria (Gombe State)	**7%**	0%
				Ethiopia (Oromia, Tigray, Amhara and SNNP)	**3%**	0%
				India (Uttar Pradesh)	**54%**	0%
						Breasts checked
				Nigeria (Gombe State)	**7%**	3 (1–5)
				Ethiopia (Oromia, Tigray, Amhara and SNNP)	**3%**	4 (2–6)
				India (Uttar Pradesh)	**54%**	28 (24–33)
						Bleeding checked
				Nigeria (Gombe State)	**7%**	1 (0–3)
				Ethiopia (Oromia, Tigray, Amhara and SNNP)	**3%**	3 (2–5)
				India (Uttar Pradesh)	**54%**	15 (11–19)
						Counselled about danger signs
				Nigeria (Gombe State)	**7%**	0%
				Ethiopia (Oromia, Tigray, Amhara and SNNP)	**3%**	2 (1–4)
				India (Uttar Pradesh)	**54%**	21 (17–26)
						Counselled about nutrition
				Nigeria (Gombe State)	**7%**	5 (3–7)
				Ethiopia (Oromia, Tigray, Amhara and SNNP)	**3%**	4 (2–6)
				India (Uttar Pradesh)	**54%**	21 (18–26)
						Counselled about family planning
				Nigeria (Gombe State)	**7%**	3 (1–5)
				Ethiopia (Oromia, Tigray, Amhara and SNNP)	**3%**	4 (2–6)
				India (Uttar Pradesh)	**54%**	17 (13–20)

POSTNATAL

Reference	Intervention	Target population	Data	Country (geographical scope)	Service contact (utilization)	Likelihood of service
Marchant T et al. 2015.	Postnatal care for newborn (Weigh newborn; check cord care; counsel caregiver on breastfeeding, thermal care and danger signs)	Women with a live birth in previous 12 months	Household surveys and frontline worker and facility surveys in selection areas in each country		PNC baby	the percent of newborns who had a post-natal check within 48 hours of birth and for whom all five post-natal processes were met
				Nigeria (Gombe State)	**4%**	0%
				Ethiopia (Oromia, Tigray, Amhara and SNNP)	**4%**	0%
				India (Uttar Pradesh)	**19%**	0%
						Weight checked
				Nigeria (Gombe State)	**4%**	**2 (1–6)**
				Ethiopia (Oromia, Tigray, Amhara and SNNP)	**4%**	2 (1–4)
				India (Uttar Pradesh)	**19%**	8 (6–11)
						Cord checked
				Nigeria (Gombe State)	**4%**	3 (1–5)
				Ethiopia (Oromia, Tigray, Amhara and SNNP)	**4%**	4 (3–7)
				India (Uttar Pradesh)	**19%**	11 (8–15)
						Body examined for danger signs
				Nigeria (Gombe State)	**4%**	3 (1–5)
				Ethiopia (Oromia, Tigray, Amhara and SNNP)	**4%**	2 (1–4)
				India (Uttar Pradesh)	**19%**	2 (1–4)
						Caregiver counselled about thermal care
				Nigeria (Gombe State)	**4%**	1 (0–3)
				Ethiopia (Oromia, Tigray, Amhara and SNNP)	**4%**	2 (1–5)
				India (Uttar Pradesh)	**19%**	1(0-3)
						Caregiver counselled about breast feeding
				Nigeria (Gombe State)	**4%**	3 (1–5)
				Ethiopia (Oromia, Tigray, Amhara and SNNP)	**4%**	5 (3–8)
				India (Uttar Pradesh)	**19%**	11 (9–15)

INFANCY

Reference	Intervention	Target population	Data	Country (geographical scope)	Service contact (utilization)	Likelihood of service	Crude coverage	Quality-adjusted coverage	user-adjusted coverage	Outcome-adjusted coverage
Nanthavong et al. 2015.	DPT vaccine	children 12-59 months	cross-sectional sero-survey in2 districts in Laos	Laos (1 province, 2 districts			Proportion vaccinated with DPT3 (based on card or caretaker's recall)			Proportion with Diphteria antibodies (% long term antibodies
							60%			63.6% (43.9)
										Proportion with tetanus antibodies (long term antibodies)
										71.2% (57.6%)
Deming MS et al. 2002.	Neonatal Tetanus	mothers of children < 1 year	MICS survey in Central African Republic 1996, follow-up of 1994-95 DHS sample. Collection of blood sample from mother of children < 1 year for testing for presence of tetanus antitoxin titres	Central African Republic	proportion of mothers who attended at least 1 ANC during last pregnancy;		proportion of mothers who were able to show vaccination card			
					**77.90%**		**57.70%**			
					proportion of mothers who delivered in a health facility/trained birth attendant		proportion of mothers protected at delivery according to history of TT vaccination			tetanus antitoxin seroprevalence proportion among mothers tested
					**63.50%**		**74.4% (67.0-81.7)**			**88.7% (83.2-94.2)**
Hayford et al. 2013.	6 indicators of measles vaccination status, (maternal report, vaccination card records, 'card + history' and EPI clinic records, immune markers in oral fluid and blood). Seroprotection in oral fluids and blood was assumed to be vaccine induced because maternal antibodies should have been non-detectable	A cross-sectional population-based study of children ages 12–16 months in Mirzapur, Bangladesh	1260 were enrolled in the study from September 2010 to January 2011	Mirzapur, Bangladesh			Maternal report of measle vaccination			Coverage of protective levels of measles IgG antibodies in Oral Fluids
							90.8% (CI: 89.0, 92.3)			57.3% (CI: 54.5, 60.1)
							EPI clinic records			Assuming 85% seroconversion among children ages 9–10 months, MCV1 coverage by OF
							88.6% (CI: 86.3, 90.6)			67.40%
							Vaccination card among card holders			Accounting for the reported sensitivity and specificity of the assay
							85.6% (CI: 83.3, 87.7)			71.50%
							card + history			Protective levels of measles IgG antibodies in blood
							84.2% (CI: 82.1, 86.2)			88.80%
Colson KE et al. 2015.	Assess difference between crude and effective coverage of Measles immunization	children under 5 years	household surveys, Dried Blood Spots tests for children 12-23 months, health center interviews	municipalities representing poorest wealth quintiles in Nicaragua and Mexican State of Chiapas			proportion of children with at least 1 documented/reported measles immunization (card-based coverage(=crude coverage), recall-based coverage, or survey-based coverage)			proportion of children with a positive DBC assay for measles antibodies (effective coverage)
				Mexico			Recall or card: 83%			68% (63%-73%)
							Recall only: 73%			
							card only: 73%			
				Nicaragua			Recall or card:85			50% (40%-61%)
							Recall only: 81%			
							card only: 82%			

INFANCY

Reference	Intervention	Target population	Data	Country (geographical scope)	Service contact (utilization)	Likelihood of service	Crude coverage	Quality-adjusted coverage	user-adjusted coverage	Outcome-adjusted coverage
Nguhiu PK, et al. 2017.	complete set of basic vaccines	children 12-23 months	Kenya DHS 2003, 2008, 2014 and Kenya SPA	Kenya			Proportion of children alive 12-23 months who received: BCG, 3 OPV, 3 DPT/HepB/HIB/PENTA, measles and 3 pneumoccal vaccines			Immunizaton rate adjusted facility-level score based on observed health worker reported availability of at least one working weighing scale and thermometer, and routinely performed processes including use of guidelines to assess and treat sick children, routine weighing, temperature taking and recording, assessment of immunization status and keeping of individual patient records
				2014			80.1 (78.5-81.7)			55.6 (53.8-57.4)
				2008/9			76.5 (72.9-80.1)			54.1 (51.6-56.6)
				2003			59.3 (55.8-62.8)			34.4 (32.1-36.7)
Khan, Z. et al. 2000.	Measles immunization	456 children in the 0-5 year age group (332 children in the 9-60 month age range)	A cross-sectional study involving the examination of children, and Hemaggltination Inhibition Test for Measles Antibody Titre. Systematic random sampling of 25% of the 1180 children 0-5 yrs living in 9 registered villages	Rural area of Aligarh District, India			Measles immunization coverage<br>64.5% (214/332)			Proportion of children with seropositivity to the measles vaccine<br>64.2% (213/332)
Mokdad, AH. et al. 2015.	MMR Immunization	Analysis of vaccination histories of children 11-59 months of age from large household surveys in Mesoamerica.	Children aged 0-59 months (coverage for children 13.5 - 59 months)	Number of Children aged 0-59 in each country survey with vaccination information			MMR coverage according to vaccination card **and** recall (%) - coverage for children 13.5-59 months		MMR coverage a**ccording to card only** considering timeliness (%) - coverage for children 13.5 - 59 months (this exludes children without vaccination cards. If the child has completed the number of required doses for [illegible]	
				El Salvador - 3457 (3110 w/ card)			90.90%		56.80%	
				Guatemala - 5191 (4311 w/ card)			84.90%		64.00%	
				Hondorus - 3022 (2620 w/ card)			93.80%		66.90%	
				Mexico - 6301 (5399 w/card)			73.50%		38.70%	
				Nicaragua - 2201 (1734 w/ card)			90.70%		52.90%	
				Panama - 2062 (1694 w/ card)			88.10%		66.20%	

CHILDHOOD

Reference	Intervention	Target population	Data	Country (geographical scope)	Service contact (utilization)	Likelihood of service	Crude coverage	Quality-adjusted coverage	user-adjusted coverage	Outcome-adjusted coverage
Smith et al. 2010.	Malaria treatment in children under-five	children under-five with fever in the two weeks preceding the survey	Household survey (305 children under-five with fever in the past two weeks)	Senegal (Tambacounda region)	careseeking from a formal provider (health post, CHW, health facility) **40%**		Presumptive treatment of fever with ACT **6%**			
Millar KR et al. 2014.	Prompt treatment; diagnostic blood test and artemisinin-based combination therapy (ACT)	Cross-sectional survey of caregivers with children < 5 years of age with fever in the last two weeks	LQAS, Mothers of Children aged 0-59 with fever in last two weeks	Sokoto and Bauchi States (northern nigeria) -	Careseeking from a formal provider 35.3%<br>% sought prompt formal care 20.9%		% who received a diagnostic blood test 7.5%<br>% who sought prompt care, and had diagnostic blood test 3.3%<br>% who received ACT 7.7%<br>% who sought prompt care, and had blood test and received ACT 0.9%			
Wangdi, K et al. 2014.	LLIN possession and use	Household population	Households survey in two districts (does not appear to have targeted any age or gender)	Four selected sub-districts of Sarpang and Samdrup Jongkhar, two historically high-incidence districts of Bhutan on the border with India.			% of household members who slept under an LLIN the night before 98.40%			% of household member testing negative for plasmodium falciparum (based on a sub-sample) 100%

CHILDHOOD

Reference	Intervention	Target population	Data	Country (geographical scope)	Service contact (utilization)	Likelihood of service	Crude coverage	Quality-adjusted coverage	user-adjusted coverage	Outcome-adjusted coverage
	Management of diarrhea	children under 5 who reported diarrhea in past 4 weeks	Kenya DHS 2003, 2008, 2014 and Kenya SPA	Kenya	%Children under 5 who had diarrhea in past 4 weeks and were given ORT or increased fluids		Proportion of children who had diarrhea in the past 4 weeks who were given the guideline recommended ORS mixture			
				2014	81.8 (80.0-83.6)		53.8 (51.2-56.4)			
				2008/9	77.2 (73.1-81.3)		38.8 (33.9-43.7)			
				2003	76.7 (73.2-80.2)		29.2 (25.4-33.0)			
Nguhiu PK et al. 2017.	careseeking for ARI,	children under 5 who reported ARI or fever in past 2 weeks	Kenya DHS 2003, 2008, 2014 and Kenya SPA	Kenya	all children with ARI/fever for whom advice on treatment was sought from a medical provider	Facility-level score based on observed/reported availability of at least 1 working weighing scale and thermometer, and routinely performed processes including use of guidelines to assess and treat sick children, routine weighing, temperature taking and recording, assessment of immunization status and keeping of individual patient records				
				2014	58.5 (56.6-60.4)	41.1 (39.5-42.7)				
				2008/9	47.7 (43.8-51.6)	33.0 (30.3-35.7)				
				2003	45.5 (42.4-48.6)	25.5 (23.7-27.3)				
	use of insecticide treated nets	all children and pregnant women	Kenya DHS 2003, 2008, 2014 and Kenya SPA	Kenya	% children and pregnant women living in household with ITN				proportion of children and pregnant women who slept under ITN during the previous night	
				2014	75.3 (73.9-76.7)				59.0 (57.4-60.6)	
				2008/9	72.6 (68.5-76.7)				53.8 (49.9-57.7)	
				2003	22 (19.8-24.2)				7.0 (5.9-8.1)	
Leslie HH et al . 2017.	Sick child treatment services: careseeking for diarrhea, fever, and symptoms of ARI	Child under-five who had experienced diarhhea, fever, or acute respiratory illness in the prior two weeks	DHS/MICS surveys linked with SPA in the past decade	8 countries: Haiti, Kenya, Malawi, Namibia, Rwanda, Senegal, Tanzania, uganda,	Under-5 care seeking for diarrhea, fever and ARI from formal provider			Effective coverage: product of crude coverage and quality score defined as the percent of 21 items performs*		
				Haiti	39.9%±2.3%			11.8%±0.4%		
				Kenya	50.8%±1.7%			22.9%±0.9%		
				Malawi	67.3%±0.7%			20.7%±0.4%		
				Namibia	61.2%±1.7%			33.0%±1.1%		
				Rwanda	41.1%±1.3%			13.2%±0.5%		
				Senegal	39.1%±1.5%			11.7%±0.5%		
				Tanzania	48.2%±1.8%			15.6%±0.6%		
				Ugandan	74.1%±1.2%			36.3%±1.2%		

* Items are: History (Inability to dring anything, normal feeding pattern, sick feeding pattern, cough or difficult breathing, diarrhea and blood in stool, fever, vomiting, convulsions, maternal HIV status, Ear problems) Routine examination (weight, Plotted weight on chart, temperature, pallor, edema of feet, mouth (thrush in IMCI)) Drug administation and immunization (Checked immunization card or immunized, Vitamin A dosage, Deworming medication); Client Education and Counseling (Explained how to administer prescribed medication, Directions for feeding, Described danger signs requiring return to facility, Scheduled/discussed return visit, Gave diagnosis)

NUTRITION

Reference	Intervention	Target population	Data	Country (geographical scope)	Service contact (utilization)	Likelihood of service	Crude coverage	Quality-adjusted coverage	user-adjusted coverage	Outcome-adjusted coverage
Aaron et al. 2017.	To assess the coverage of food fortification programs as well as to determine whether vulnerable or at-risk population groups benefited from the respective programs between 2013-2015	Households and women in reproductive age 15-49, and their children aged 0-24 months		**8 countries**			Proportion of households that consumes the vehicle		Proportion of households that used the food vehicle which was fortified	
				**Wheat Flour**						
				Cˆote d_Ivoire (Abidjan)			54.7 (50.1, 59.6)		NA	
				India (Rajasthan)			83.2 (79.5, 86.5)		6.3 (4.8, 7.9)	
				Nigeria (Kano)			83.9 (81.5, 86.3)		22.7 (20.0, 25.5)	
				Nigeria (Lagos)			14.2 (11.8, 16.5)		5.4 (3.8, 6.9)	
				Senegal			81.8 (76.2, 86.6)		51.2 (44.7, 57.2)	
				South Africa (Eastern Cape)			25.2 (16.3, 34.1)		16.3 (10.0, 23.7)	
				South Africa (Gauteng)			4.3 (1.8, 7.6)		0.8 (0.0, 2.3)	
				Tanzania			51.5 (44.5, 58.5)		33.1 (27.5, 38.7)	
				Uganda			11.2 (7.7, 14.7)		8.5 (5.7, 11.4)	
				**Maize Flour**						
				Nigeria (Kano)			77.1 (74.4, 79.9)		1.7 (0.9, 2.6)	
				Nigeria (Lagos)			12.2 (10.0, 14.4)		0.2 (0.0, 0.5)	
				South Africa (Eastern Cape)			98.7 (96.5, 100.0)		86.8 (80.0, 92.4)	
				South Africa (Gauteng)			95.6 (90.4, 98.6)		77.4 (69.8, 94.9)	
				Tanzania			93.0 (89.7, 96.4)		2.5 (1.3, 3.7)	
				Uganda			91.8 (87.7, 96.0)		6.5 (3.3, 9.7)	
				**Edible oil**						
				Bangladesh			100			
				Cˆote d_Ivoire (Abidjan)			98.5 (97.5, 99.3)		98.0 (97.0, 99.0)	
				India (Rajasthan)			100		24.3 (21.1, 27.9)	
				Nigeria (Kano)			98.4 (97.6, 99.2)		7.6 (5.9, 9.4)	
				Nigeria (Lagos)			98.6 (97.8, 99.3)		7.2 (5.5, 8.9)	
				Senegal			97.8 (96.3, 99.1)		34.1 (29.1, 40.7)	
				Tanzania			96.2 (93.2, 99.2)		53.6 (46.4, 60.8)	
				Uganda			89.9 (85.9, 94.0)		54.4 (48.3, 60.4)	
Aaron et al. 2016.	To assesse the coverage achieved by two sales-based approaches to distributing a complementary food supplement (KOKO Plus™) to infants and young children in Ghana.	Children aged between 6 and 24 months and their principal caregivers (defined as the person who provides most care for the child and gives the child most meals on most days).		**Ghana**			Proportion of children who have ever been fed the product		Proportion of children that have fed the fortified product at least once in the past 7 days	
				Round 1 (Month 3)			94.4 (89.7, 98.1)		88.3 (81.1, 97.4)	
				Round 2 (Month 10)			92.0 (82.7, 98.7)		83.1 (73.4, 93.1)	
				Round 3 (Month 14)			84.4 (77.6, 89.9)		61.9 (53.2, 69.9)	
Leyvraz M et al. 2016.	Utilization of Fortified Take-Home Rations among Children 6–35 Months	Children age 6-35 months	Household survey (1077 children)	India (Telangana)	% of childrn whose cargiver ever received the ration 86.8%				% of children who always consume the ratio 57.2%	

NUTRITION

Reference	Intervention	Target population	Data	Country (geographical scope)	Service contact (utilization)	Likelihood of service	Crude coverage	Quality-adjusted coverage	user-adjusted coverage	Outcome-adjusted coverage
Aaron GJ et al. 2016.	To assess statewide household coverage of fortified staple foods: atta wheat flour, edible oil and salt in Rajhastan.	All households that had a caregiver with a child under 2 years. Caregiver defined as biological mother or person who cared for and gave child most meals on most days.	household survey in state of Rajhastan	India: State of Rajhastan			proportion of households that consumed the vehicle<br><br>Atta wheat flour: 83.2%<br><br>edible oil: 100%<br><br>salt: 100% .	proportion of households that consumed the fortified vehicle<br><br>Atta wheat flour: 6.3%<br><br>edible oil: 24.3%<br><br>salt : 84.7%	proportion of households that consumed adequately fortified vehicle<br><br>Atta wheat flour: 0.1%<br><br>edible oil: NA<br><br>salt: 66.3%	
Gebremedhin S et al. 2014.	coverage, compliance and factors associated with use of of prenatal iron supplementation	Women who gave birth in last year preceding survey	2 community-based surveys	8 rural districts of Ethiopia			Proportion of women who were given/prescribed iron supplements during pregnancy (The paper did not report ANC1)<br><br>35.40%		proportion of women who took supplements for >90 consecutive days<br><br>3.50%	
Leyvraz M et al. 2016.	coverage of program of fortification of a locally-produced fortified complementary food	children under 2 years	cross sectional cluster-based household survey of caregivers of children under 2 years: September-October 2014	Abidjan, Cote d'Ivoire			"contact coverage": proportion of caregivers that ever fed the child the product<br><br>37.8% (33.3-42.5)		"partial coverage": proportion of caregivers that fed the child the product in past month<br><br>8.8% (6.4-11.9)	"effective coverage": proportion of caregivers that fed the child the product in last 7 days<br><br>4.6% (2.9-7.2)
Leyvraz M et al. 2017.	Review of 11 coverage surveys of IYCF programs distributing or selling fortified complementary foods or micronutrient powders in 5 countries	children under 59 months	11 surveys conducted between 2013-2015	Ghana, Cote d'Ivoire, India, Bangladesh, Vietnam.<br>overall:			Use of the product at least 1 time.<br>22.6%-94.4%		Regular use aligned with program-specific goals<br>0.8%-88.3%	
		children 6-59 months		Bangladesh Survey 1.1A			23.5% (20.8-26.7)		2.1% (1.4-3.2)	
				Bangladesh Survey 1.2A			36.8 % (33.4-40.9)		3.9% (2.7-5.5)	
				Bangladesh Survey 2			26.3 % (23.6-28.6)		0.8% (0.4-1.2)	
		children 0-23 months		Cote d'Ivoire endline			37.5% (32.8-42.5)		4.6% (2.9-7.2)	
		children 6-23 and children 0-23 months		Ghana Survey 1.1B			94.4% (89.7-98.1)		88.3% (81.1-94.6)	
				Ghana Survey 1.2B			92.0% (82.7-98.7)		83.1% (73.4-93.1)	
				Ghana Survey 1.3			84.4% (77.6-89.9)		61.9 (53.2-69.9)	
				Ghana Survey 2.1			23.5% (19.0-28.5)		15.3 (11.3-19.8)	
				Ghana Survey 2.2			52.8% (47.7-58.9)		9.4% (6.7-12.4)	
		children 0-35 months		India endline			86.8% (73.1-94.1)		57.2% (48.2-65.8)	
		children 6-59 months		Vietnam endline			22.6% (17.4-28.2)		12.5% (8.3-16.8)	

NUTRITION

Reference	Intervention	Target population	Data	Country (geographical scope)	Service contact (utilization)	Likelihood of service	Crude coverage	Quality-adjusted coverage	user-adjusted coverage	Outcome-adjusted coverage
Nguhiu PK et al. 2017.	Breastfeeding during first 6 months	children 0-5 months	Kenya DHS 2003, 2008, 2014 and Kenya SPA	Kenya			children 0-5 months who were breastfed in last 24 hours		children 0-5 months who were exclusively breastfed in last 24 hours	
				2014			99.6 (99.2-100)		71.6 (67.7-75.5)	
				2008/9			99.1 (98.4-99.8)		46.1 (41.0-51.2)	
				2003			99.8 (99.6-100)		68.0 (63.6-72.4)	
Nguyen M et al. 2016.	Home fortification with micronutrient power (MNP) sachets sold at at 337 Health Centers	Children 6-59 months.	Cross-sectional survey in 32 communities for caregivers (n=962) and 120 health staff. Stratified multistage cluster sampling was used to select caregivers.	4 provinces (Thai Nguyen, Hai Phon, Quang Nam, Ca Mau) in Vietnam	Caregivers who have visited the health centers in past year		Caregivers who were counseled on Home fortification with MNP		Consumption of 3 or more sachets/week (effective coverage)	
					42.0%		26.8%		11.5%	
					Caregivers who ever tried once the MNP to ther child				Consumption of at least 1 sachet/week (partial coverage)	
					22.6%				21.7%	
Engle-Stone R et al. 2015.	To assess effective coverage of Vitamin A intake (either through a vehicle of fortified food (refined vegetable oil, bouillon cube, maize, or micronutrient powders, or high dose VA capsules or deworming tablets during child health days, or indirectly through breastmilk for children still breastfeeding)	women 15-49 years and children 6-59 months (breastfeeding and non-breastfeeding)	24-hour dietary recall collected in national micronutrient survey 2009	Cameroun: 3 macro- regions: South, North and Yaounde/Douala	proportion of children 6-59 months and women exposed to an intervention ("REACH") - % are for South, North and Yaounde regions respectively	proportion of children who are deficient and receive the program ("COVERAGE") (South, North and Yaounde, respectively by program)	Coverage: Proportion of individuals who are deficient (or have inadequate intake) and are exposed to an intervention [Inadequacy was defined as the proportion of the population with usual VA intakes below the estimated average [illegible]			proportion of population that has inadequate intake at baseline and achieves sufficiency as a result of a given intervention(s) (effective coverage by intervention) % are for South, North and Yaounde respectively
					CHILDREN 6-59:		% not reported, but graphed (no label values) report approximated value from graph?. See fig2			% not reported, but graphed (no label values) report approximated value from graph?. See fig2
					Vitamin A Supplement		Vitamin A Supplement			Vitamin A Supplement
				South	64%		18%			15%
				North	89%		38%			40%
				Younde/Douala	58%		25%			20%
					Deworming					Deworming
				South	65%		15%			5%
				North	79%		32%			2%
				Younde/Douala	81%		22%			3%
					Micronutrient powder					Micronutrient powder
				South	64%		15%			12%
				North	89%		35%			33%
				Younde/Douala	58%		25%			18%
					Refined Oil (44% target level)					Refined Oil (44% target level)
				South	39%		11%			12%
				North	56%		25%			10%
				Younde/Douala	79%		28%			27%
					Bouillon cube					Bouillon cube
				South	92%		28%			12%
				North	86%		38%			12%
				Younde/Douala	90%		32%			18%
					Maize biofortification					Maize biofortification
				South	41%		10%			5%
				North	45%		20%			10%
				Younde/Douala	34%		15%			4%

APPENDIX - 3 SYNTHESIS OF METHODS

			Target population		Crude coverage (or service contact)		Quality or quality-adjusted coverage			Analytic methods	
#	Paper	Intervention	Indicator	Data Source	Indicator	different from data source for target population	Indicator	Indicator type	Data source, if different from data source for target population	Linkage strategy	Variance calculation for quality-adjusted coverage
1	Aaron et al. 2016.	Child nutrition: Food supplement	Child age, poverty, poor diet diversity, suboptimal feeding practices	Household survey (program-specific)	Caregiver has heard of product, child ever fed product		Child fed product in previous 7 days	Binary		Individual data only	Blocked weighted bootstrap
2	Aaron GJ et al. 2016.	Child nutrition: fortified foods	Child age, poverty, poor diet diversity, suboptimal feeding practices, rural location	Household survey (program-specific)	Consumption of vehicle, consumption of fortifiable vehicle		Consumption of fortified vehicle, consumption of adequately fortified vehicle	Binary	Food specimens collected during survey, assessed	Household, assume food intake proportional to need	Blocked weighted bootstrap
3	Aaron et al. 2017.	Nutrition: fortified foods	Poverty, woman's dietary diversity, rural residence	Household survey (FACT)	Consumption of vehicle, consumption of fortifiable vehicle		Consumption of fortified vehicle	Binary	Brand ID, household or market collection -> lab assessment	Household to market as needed	Bootstrap
4	Agha S et al. 2016.	Antenatal care	Women pregnant in past 2 years	Household survey (DHS-like tool)	ANC visit		Content: sum from 0 to 6 of self-report of blood pressure measurement, blood test, urine test, weight, iron, tetanus toxoid vaccination. Separately, timing: first trimester ANC	Scale		Individual data only	None
5	Baker et al. 2015b	Antenatal care	Women with recent live birth	Household survey (EQUIP)	ANC attendance - (twice for anemia)		ANC at a clinic with available testing (syphilis, anemia, HIV); ANC at facility with test and self-report of test	Binary	Facility survey (census in the area)	Ecological, stratified by facility level	None
6	Baker et al. 2015a.	Antenatal care: syphilis screening	Women with live birth within 12 months - pregnancy	Household survey (EQUIP)	1 ANC visit		ANC at a clinic with available testing (syphilis); ANC at facility with test and self-report of test	Binary	Facility survey (census in the area)	Ecological, stratified by facility level	None
		Antenatal care: pre-eclampsia screening	Women with live birth within 12 months - pregnancy	Household survey (EQUIP)	3 ANC visits		ANC at a clinic with available sphygmomanometer; ANC at facility with sphyg and self-report of BP testing	Binary	Facility survey (census in the area)	Ecological, stratified by facility level	None
		Labor and delivery: use of partograph	Women with live birth within 12 months - delivery	Household survey (EQUIP)	Birth in health facility		Health facility with partograph available, facility where health workers report using partograph	Binary	Facility survey (census in the area)	Ecological, stratified by facility level	None
		Labor and delivery: AMTSL	Women with live birth within 12 months - delivery	Household survey (EQUIP)	Birth in health facility		Health facility with sterile syringe, needs, oxytocin or ergometrine, facility at which health worker reported giving oxytocic agent	Binary	Facility survey (census in the area)	Ecological, stratified by facility level	None
		Postpartum care	Women with live birth within 12 months - delivery	Household survey (EQUIP)	Birth in health facility		Health facility offering PPC with iron supplements; facility plus report of check within 48 hours	Binary	Facility survey (census in the area)	Ecological, stratified by facility level	None
7	Colson KE et al. 2015.	Immunization	Children 1 -2 yo	Household survey (Salud Mesoamerica)	Vaccination on health card / vaccination reported by caretaker		Positive for measles IgG antibodies	Binary	DBS via household survey	Individual data only	Standard errors adjusted for survey design

8	Deming MS et al, 2002.	Antenatal care: tetanus toxoid	Mothers of children < 1	Household survey (MICS)	TT doses during last pregnancy		Tetanus antitoxin titers	Binary	DBS via household survey	Individual data only	Standard errors adjusted for survey design
9	Engle-Stone R. et al. 2015.	Vitamin A intake	Children 6 to 59 months and women 15-49 months	National survey of micronutrient status and dietary intake in 2009	Prortion of children exposed to an intervention (VA intake through VA supplementation, deworming, micronutrients) who had biochemically defined VA deficiency		proportion of the population that has inadequate intake at baseline and then ''achieves sufficiency'' as a result of a given intervention or set of interventions (based on similations).	Binary		No linking	based on similation of effect of interventions on VA intake and recalculcation of prevalence of inadequate intake of VA
10	Gakidou et al 2008.	Cervical cancer screening	Women 25 to 64	Household survey (WHS)	Pelvic exam ever		Self report of pelvic exam and pap smear in past 3 years	Binary		Individual data only	Standard errors adjusted for survey design
11	Gebremedhin S et al. 2014.	Antenatal care: iron use	Currently pregnant	Household survey (program-specific)	ANC visit		Anemia, self-reported iron supplement use	Binary	Blood sample via household survey	Individual data only	None
		Antenatal care: iron use	Women giving birth in past years	Household survey (program-specific)	ANC visit		Self-reported iron supplement use	Binary		Individual data only	Standard errors adjusted for survey design
12	Hayford et al. 2013.	Immunization	Children 12 - 16 months	Household survey	Card / report of vaccination		IgG antibodies	Binary	Oral and blood samples from household survey	Individual data only	Standard errors adjusted for survey design
13	Heredia-Pi, et al. 2016.	Antenatal care	Women with live birth 2006 - survey	Household survey (ENSANUT)	Skilled ANC		Timely, sufficient, appropriate ANC - 8 items of appropriate ANC from self report, binary indicator based on top quintile	Binary		Individual data only	Standard errors adjusted for survey design
14	Hodgins et al, 2014.	Antenatal care: hypertensive disease	Women delivering in past 2 years	Household survey (DHS)	ANC visit, 4 ANC visits		Self-reported blood pressure measurement	Binary		Individual data only	
		Antenatal care: tetanus toxoid	Women delivering in past 2 years	Household survey (DHS)	ANC visit, 4 ANC visits		Self-reported at least two doses tetanus toxoid vaccine	Binary		Individual data only	
		Antenatal care	Women delivering in past 2 years	Household survey (DHS)	ANC visit, 4 ANC visits		Self-reported early ANC (first 4 months gestation)	Binary		Individual data only	
		Antenatal care	Women delivering in past 2 years	Household survey (DHS)	ANC visit, 4 ANC visits		Self-reported urine test	Binary		Individual data only	
		Antenatal care	Women delivering in past 2 years	Household survey (DHS)	ANC visit, 4 ANC visits		Self-reported counseling on danger signs	Binary		Individual data only	
		Antenatal care: HIV	Women delivering in past 2 years	Household survey (DHS)	ANC visit, 4 ANC visits		Self-reported HIV counseling and testing	Binary		Individual data only	
		Antenatal care: iron use	Women delivering in past 2 years	Household survey (DHS)	ANC visit, 4 ANC visits		Self-reported use of iron/folate for at least 90 days	Binary		Individual data only	
		Antenatal care: malaria prevention	Women delivering in past 2 years	Household survey (DHS)	ANC visit, 4 ANC visits		Self-reported two doses of sulfadoxine/pyramethamine	Binary		Individual data only	
		Antenatal care	Women delivering in past 2 years	Household survey (DHS)	ANC visit, 4 ANC visits		Self-reported receipt of all interventions	Binary		Individual data only	Standard errors adjusted for survey design
15	Kanyangarara et al. 2017.	Antenatal care: tetanus toxoid	Women with live birth within 3 years	Household survey (DHS)	4 ANC visits		Valid TT vaccine	Binary	Facility surveys (sample, SPA and SARA)	Ecological, stratified by facility level, management, and location (urban / rural)	None - some sensitivity analyses
		Antenatal care: malaria prevention	Women with live birth within 3 years	Household survey (DHS)	4 ANC visits		Valid sulfadoxine/pyramethamine	Binary	Facility surveys (sample, SPA and SARA)	Ecological, stratified by facility level, management, and location (urban / rural)	None - some sensitivity analyses
		Antenatal care: syphilis screening	Women with live birth within 3 years	Household survey (DHS)	4 ANC visits		Valid syphilis test and medication	Binary	Facility surveys (sample, SPA and SARA)	Ecological, stratified by facility level, management, and location (urban / rural)	None - some sensitivity analyses
		Antenatal care: hypertensive disease	Women with live birth within 3 years	Household survey (DHS)	4 ANC visits		Valid dipstick, blood pressure apparatus, magnesium sulfate	Binary	Facility surveys (sample, SPA and SARA)	Ecological, stratified by facility level, management, and location (urban / rural)	None - some sensitivity analyses
		Antenatal care: iron use	Women with live birth within 3 years	Household survey (DHS)	4 ANC visits		Valid iron or iron folate	Binary	Facility surveys (sample, SPA and SARA)	Ecological, stratified by facility level, management, and location (urban / rural)	None - some sensitivity analyses

16	Khan, Z. et al. 2000.	Immunization	Children 0 - 5	Household survey	Reported / card vaccination status		IgG antibodies	Binary	Blood sample via household survey	Individual data only	None
17	Kiwanuka , HD et al. 2017.	Antenatal care: iron use	Women pregnant in past year	Household survey (EQUIP)	Health facility w/in 5 km	Geospatial data on facility location	Availability of iron folate, self report of >90 day consumption	Binary	Facility survey (census in the area)	Individual to facility - nearest public facility by linear distance	None
		Antenatal care: malaria prevention	Women pregnant in past year	Household survey (EQUIP)	Health facility w/in 5 km	Geospatial data on facility location	Availability of sulfadoxine/pyramethamine, women reporting at least two doses SP	Binary	Facility survey (census in the area)	Individual to facility - nearest public facility by linear distance	None
		Antenatal care: HIV	Women pregnant in past year	Household survey (EQUIP)	Health facility w/in 5 km	Geospatial data on facility location	Availability of HIV test kits, women reporting getting tested and receiving results	Binary	Facility survey (census in the area)	Individual to facility - nearest public facility by linear distance	None
		Antenatal care: syphilis screening	Women pregnant in past year	Household survey (EQUIP)	Health facility w/in 5 km	Geospatial data on facility location	Availability of syphilis tests, women reporting syphilis testing and receiving results	Binary	Facility survey (census in the area)	Individual to facility - nearest public facility by linear distance	None
18	Kyei NN et al. 2012.	Antenatal care	Women with live birth within 5 years	Household survey (DHS)	ANC visit		Health facility service provision (services, staff, testing) and woman's report of ANC interventions	Binary and categorical reported for service provision, Categorical for quality based on # of interventions reported	Facility survey (census)	None - facility and population data reported separately	NA
19	Larson E, et al. 2017.	Obstetric care	Women who had a delivery of a child within the year prior ot the survey	Household interviews	Births in health facility		Five dimension of quality of care: 1) facility infrastructure; 2) availability of equipment, supplies and medicines; 3) health worker knowledge and competence; 4) provision of routine obstetric services; and 5) provision of emergency obstetric and newborn services	Scale	Facility audit and provider interviews	Ecological	None
20	Larsson EC et al. 2012.	Antenatal care: HIV	Women pregnant at study enrollment	Population cohort (DSS)	ANC visit		HIV testing during ANC	Binary	Facility record review	Individual to facility - record linked	None
21	Leslie HH et al . 2017.	Family planning	Women with contraceptive need	Household survey (DHS, MICS)	Use of modern contraceptive		Adherence to evidence based guidelines	Scale	Facility survey (census and sample)	Ecological, stratified by subnational region	Direct calculation
		Antenatal care	Women with live births within 2 years	Household survey (DHS, MICS)	1 ANC, 4 ANC		Adherence to evidence based guidelines	Scale	Facility survey (census and sample)	Ecological, stratified by subnational region	Direct calculation
		Sick child	Children < 5 with recent illness	Household survey (DHS, MICS)	Use of formal health care		Adherence to evidence based guidelines	Scale	Facility survey (census and sample)	Ecological, stratified by subnational region	Direct calculation
22	Leyvraz M et al. 2016.	Child nutrition: fortified foods	Children 0 - 23 months	Household survey (program-specific)	Caregiver has heard of product, child ever fed product		Child fed product in previous 7 days	Binary		Individual data only	Blocked weighted bootstrap
23	Leyvraz M et al. 2016.	Child nutrition: fortified foods	Children 0 - 35 months	Household survey (program-specific)	Caregiver has heard of product, child ever fed product		Child fed product in previous 7 days	Binary		Individual data only	Blocked weighted bootstrap
24	Leyvraz M et al. 2017.	Child nutrition: fortified foods	Children under 5, varying ranges by country	Household survey (program-specific)	Caregiver has heard of product, child ever fed product		Child fed product with adequate quantity with adequate frequency	Binary		Individual data only	Blocked weighted bootstrap

25	Lozano R et al. 2006.	Services delivered to premature babies	Births in Ministry of Health database	Ministry of Health routine data	Not defined		Effective coverage of services delivered to premature babies is defined as the proportion of the maximum possible health gain a moderately premature baby could expect to receive that is actually delivered	Proportion		None	Direct calculation
26	Marchant T et al. 2015.	Antenatal care	Women with live birth within 12 months	Household survey (program-specific)	1 ANC visit		All 8: Weight & height, BP, urine and blood tests; counselling for breastfeeding, danger signs, birth preparedness	Binary		Individual data only	Standard errors adjusted for survey design
		Labor and delivery: AMTSL	Women with live birth within 12 months	Household survey (program-specific)	Birth attended by SBA		Active management of third stage of labor (AMTSL)	Binary	Healthworker interviews	Cluster	Not specified
		Postpartum care	Women with live birth within 12 months	Household survey (program-specific)	Postpartum check with 48 hours		All 5: breasts and bleeding; counseling on danger signs, nutrition, family planning	Binary		Individual data only	Standard errors adjusted for survey design
		Postnatal care	Newborns born alive in past 12 months	Household survey (program-specific)	Postnatal check within 48 hours		All 5: Weigh newborn, check cord, counsel on breastfeeding, thermal care, danger signs	Binary		Individual data only	Standard errors adjusted for survey design
27	Martinez, S. et al. 2011.	Antenatal care; Skilled attendant at birth; Exclusive breastfeeding, child vaccination; child growth monitoring	Children under under five years; women who had a pregnancy in last 5 years (last birth)	Household survey (DHS) for all countries except Chile and Costa Rica where Administrative information systems were used	Proportion of target population that receive the intervention based on self report		Crude coverage adjusted for quality based on biomarkers, self-report, process measures, mortality rate).	Binary		None	None
28	Millar KR et al. 2014.	Sick child	Children 0- 59 months with recent fever	Household survey LQAS	Treatment sought outside home		Diagnostic test, medication, medication type and timing	Binary		Individual data only	None
29	Mokdad, AH. et al. 2015.	Immunization	Children 0 to 5	Household survey (Salud Mesoamerica)	Reported / card vaccination status (1+ MMR dose)		Card based timely vaccination	Binary		Individual data only	None
30	Nanthavong et al. 2015.	Immunization	Children 12 - 59 months	Household survey	Card / report of vaccination		Anti-diphtheria antibody levels	Binary	Blood sample via household survey	Individual data only	Standard errors adjusted for survey design
		Immunization	Children 12 - 59 months	Household survey	Card / report of vaccination		Anti-tetanus antibodies	Binary	Blood sample via household survey	Individual data only	Standard errors adjusted for survey design
31	Ndyomugyenyi R et al. 2010.	Antenatal care: malaria prevention	Women giving birth in past month	Household survey	ANC visit		Receipt of IPTp-SP	Binary		Individual data only	None
		Family planning	Women 15-49 able to get pregnant	Household survey (DHS)	Use of modern contraceptive		Facility score: privacy, visual aids and records, commodity management practices	Scale	Facility survey (sample, SPA)	Ecological, stratified by subnational region	Taylor series expansions
		Antenatal care	Women 15 - 49 with 1+ child in past 5 years	Household survey (DHS)	4 ANC visits		BP, urine, blood, complications, iron, parasites	Scale		Individual data only	Taylor series expansions
		Delivery and PNC	Women 15 - 49 with 1+ child in past 5 years	Household survey (DHS)	Birth attended by SBA		Facility score: rooming in, weighing, examination, BCG, other indicators	Scale	Facility survey (sample, SPA)	Ecological, stratified by subnational region	Taylor series expansions
		Breastfeeding	Children 0 - 5 months	Household survey (DHS)	Breastfed in past 24 hours		Exclusive breastfeeding past 24 hours	Binary		Individual data only	Taylor series expansions

32	Nguhiu PK et al. 2017.	Immunization	Children 12 - 23 months	Household survey (DHS)	Complete vaccination per national guideline		Facility score: scale, thermometer, use of guidelines for sick kids, routine weighing, temp taking, recording, checking of vaccination status, keeping individual records	Scale	Facility survey (sample, SPA)	Ecological, stratified by subnational region	Taylor series expansions
		Sick child	Children under 5 with diarrhea past two weeks	Household survey (DHS)	Given ORT or increased fluids		Self report on receipt of recommended ORS	Binary		Individual data only	Taylor series expansions
		Sick child	Children under 5 with resp symptoms or fever	Household survey (DHS)	Use of formal health care		Facility score: scale, thermometer, use of guidelines for sick kids, routine weighing, temp taking, recording, checking of vaccination status, keeping individual records	Scale	Facility survey (sample, SPA)	Ecological, stratified by subnational region	Taylor series expansions
		Malaria prevention	Children and pregnant women	Household survey (DHS)	Household that owned ITN		Self report that individual actually slept under ITN night before	Binary		Individual data only	Taylor series expansions
33	Nguyen M et al. 2016.	Child nutrition: fortified foods	Children 6 to 60 months old	Household survey (FACT)	Caregiver has heard of product, child ever fed product		Children had product in past week, children had 3 packets in past week	Binary		Individual data only	None
34	Smith et al. 2010.	Sick child	Children under 5 with fever past 2 weeks	Household survey	Use of formal health care		Self report on timing of treatment seeking, use of RDT, dose and duration of anti-malarials	Binary		Individual data only	None
35	Thapa et al. 2016.	Antenatal care: hypertensive disease	Women giving birth in past 6 months	Household survey	ANC visit		Self-reported calcium use	Binary		Individual data only	None
36	Wangdi, K et al. 2013.	Malaria prevention	Household	Household survey	LLIN ownership		LLIN use, upkeep	Binary		Individual data only	None
